# NFIL3/Tim3 axis regulates effector Th1 inflammation in COPD mice

**DOI:** 10.3389/fimmu.2024.1482213

**Published:** 2024-11-01

**Authors:** Junyi Ke, Shu Huang, Zhixiong He, Siyu Lei, Shiya Lin, Yinying Li, Qiuming Li, Hui Huang, Hongchun Huang, Huajiao Qin, Minchao Duan

**Affiliations:** ^1^ Guangxi Medical University, Nanning, China; ^2^ The Second Affiliated Hospital of Guangxi Medical University, Nanning, China; ^3^ Wuming Hospital of Guangxi Medical University, Nanning, China

**Keywords:** Tim3, Th1, COPD, NFIL3, flow cytometry, single-cell sequencing

## Abstract

**Background:**

IFN-γ+CD4+ cells (type 1 helper T cells, Th1) represent a critical component of the inflammatory environment in the lungs of chronic obstructive pulmonary disease (COPD). Identifying influencing factors related to COPD-associated Th1 cells will enhance our understanding of the inflammatory mechanisms involved and facilitate the development of targeted interventions.

**Method:**

We describe T-cell immunoglobulin and mucin-domain containing-3 (Tim3) as a key gene regulating COPD-associated Th1 cells through single-cell sequencing, flow cytometry and knockout mice.

**Results:**

Our findings indicate that Havcr2 expression gradually increases during CD4+ T cell activation in COPD mice, with Tim3 being highly expressed on both CD4+ T cells and Th1 cells. Notably, the knockout of HAVCR2 further promotes the infiltration of CD4+ T cells and the expression of IFN-γ in the lungs, resulting in a more severe emphysema phenotype, although it does not significantly affect TNF-α expression. Additionally, NFIL3, an upstream regulator of Tim3, is also highly expressed in the CD4+ T cells of COPD mice. Mice with NFIL3 knockout exhibit phenotypes similar to those of HAVCR2 knockout mice, along with a significant downregulation of Tim3 expression. *In vitro*, we simulated the activation process by polarizing primary CD4+ Tn cells from COPD mice and observed that NFIL3/Tim3 expression was significantly upregulated following Th1 polarization.

**Conclusion:**

Our study demonstrates that the NFIL3/Tim3 axis plays a role in Th1 imbalance in the lungs of COPD by inhibiting Th1 differentiation.

## 1 Introduction

Chronic obstructive pulmonary disease (COPD) is a heterogeneous condition characterized by irreversible airflow limitation (1). In recent years, the incidence of COPD has risen, establishing it as one of the major diseases threatening human health (2). The primary pathological manifestations of COPD include chronic inflammation and emphysema. Inflammatory cell infiltration and alveolar cell apoptosis underlie a series of pulmonary pathological changes associated with COPD (3). It is widely accepted that smoking is the principal cause of COPD, with approximately 90% of COPD patients having a history of smoking (4). The continuous exposure to tobacco smoke perpetuates damage to the airways and alveoli, resulting in the destruction of lung structures and inflammatory infiltration (5). The COPD animal model, developed through whole-body passive smoking, has been validated in C57BL/6J mice. This model effectively simulates the lung inflammation and structural changes characteristic of COPD, and has become a mainstream approach in the pathological study of the disease (6).

Th1 cells, the predominant effector CD4+ T cell subtype, are a significant source of IFN-γ and play a crucial role in the body’s adaptive immunity (7). Th1 cells are closely associated with chronic immune inflammation in COPD. Chronic tobacco exposure can activate the expression of transcription factors T-bet and STAT4 in CD4+ T cells, which are critical for Th1 differentiation (8, 9). In the stable phase of COPD, an IFN-γ dominant T cell cytokine pattern is observed, characterized by a notable increase in Th1 cell numbers and elevated IFN-γ expression (10, 11). However, the specific regulatory mechanisms of Th1 cells in COPD remain unclear. Our previous studies established the impact of T cell immunoglobulin mucin molecule 3 (Tim3) on CD8+ T cells and Th17 cells within the COPD lung microenvironment (12, 13). Nevertheless, the effect of Tim3 on Th1 cells in the context of COPD is currently unknown. Therefore, this study primarily aims to explore whether Tim3 influences Th1 inflammation in COPD.

Tim3 is a crucial immune checkpoint encoded by HAVCR2, capable of binding to ligands such as Gal-9 to generate inhibitory signals. It primarily maintains immune balance by regulating the functional impairment of immune cells and serves as a significant indicator of the body’s immune tolerance (14). Numerous studies have confirmed that Gal-9/Tim3 signaling regulates Th1 apoptosis, influences the production of Th1-derived cytokines, and is closely associated with autoimmune cerebrospinal meningitis and type I diabetes (15). Nuclear factor interleukin 3 (NFIL3) is a mammalian basic leucine zipper transcription factor encoded by NFIL3, which is widely expressed across various mammalian tissues (16). Additionally, NFIL3 is recognized as an important transcriptional repressor protein (17), closely linked to the development and differentiation of immune cells (18). Zhu et al. (19) discovered that functional changes in Tim3+ T cells can be induced in an NFIL3-dependent manner. However, the involvement of the NFIL3/Tim3 signaling axis in tobacco-induced Th1 immune inflammation in the lungs remains unexplored.

In this study, we simulated the lung microenvironment of COPD patients using single-cell sequencing data from COPD mice and tobacco-induced COPD mouse models, examined the expression patterns of Tim3 and NFIL3 in the lungs, and investigated the effects of the NFIL3/Tim3 axis on Th1 differentiation in COPD mice. These findings enhance our understanding of Th1 inflammation in COPD.

## 2 Materials and methods

### 2.1 Mice and cigarette smoke exposure

Havcr2 knock out and Nfil3 knock out mice on a C57BL/6J background were obtained from the Shanghai Model Organisms Center in Shanghai, China. Briefly, the Cas9 mRNA and gRNA targeting HAVCR2 and NFIL3 were synthesized through *in vitro* transcription. Subsequently, the Cas9 mRNA and gRNA were microinjected into the fertilized eggs of C57BL/6J mice to generate F0 generation mice, which were then identified by PCR. The F0 generation mice were mated with C57BL/6J mice to produce F1 generation knockout mice (13). Wild-type C57BL/6J mice, aged 6-8 weeks, were purchased from the Experimental Animal Center of Guangxi Medical University in Nanning, China. All mice were housed in sterile cages, maintained in a 12:12 h light-dark environment, and provided with sterile food and water ad libitum. The animal studies were approved by the Experimental Animal Center of Guangxi Medical University.

To establish the pathological model of tobacco-exposed emphysema (20), mice were exposed to cigarette smoke in a closed room (0.75m^3^) where 5 cigarettes were lit each time, with a 30-minute smoke-free interval, 4 times a day, 5 days a week, for 24 weeks. Mice showed tolerance to cigarette smoke exposure without any apparent signs of toxicity. Control group mice were housed in a smoke-free air environment for the same duration. Mice were euthanized using pentobarbital anesthesia and analyzed 24 hours after the final exposure to cigarette smoke.

### 2.2 Mice lung and spleen tissue processing

The methodology for collecting and processing mouse lung tissue followed established procedures (22). Mice were euthanized using pentobarbital anesthesia, and the lungs were flushed with PBS to eliminate intravascular red blood cells. The lung tissue was then stored in PBS at 4°C and processed within one hour. After mincing the lungs, they were digested in RPMI 1640 medium with type IV collagenase, followed by mechanical trituration and filtration through a 70 μm cell strainer. The resulting cells were washed, centrifuged, and resuspended in PBS. Similarly, mouse spleen tissue was collected, ground, filtered, and treated with red blood cell lysis buffer before being centrifuged and resuspended in PBS.

### 2.3 Histology and morphometry of mice lungs

Ten random fields at a magnification of 200× were assessed by two blinded pathology investigators using Image J software to evaluate emphysema extent. The mean alveolar lining interval (Lm) measurement method described previously (24) was used for assessment. Lm was measured independently by two researchers in a blinded fashion.

### 2.4 Flow cytometry

Surface proteins and cytokines of lung T cells were analyzed by flow cytometry using antibodies against CD3, CD4, IFN-γ, TNF-α, Tim3, NFIL3, CD44, CD62L, and T-bet. Cells were processed following standard protocols: surface proteins were stained, followed by cell stimulation with 25ng/ml phorbol-myristate-acetate (PMA) and 1ng/ml ionomycin for 4 hours at 37°C, 5% CO_2_. Subsequently, cells were stained for surface proteins, treated with fixation/permeabilization solution, and then stained for cytokines. Each staining session lasted approximately 30 minutes. Finally, cells were washed with Perm/Wash Buffer, resuspended in PBS, and analyzed using a BD FACS Canto II flow cytometer, with data processed using Flowjo software.

### 2.5 Cell isolation and culture

Naive CD4+ T cells were isolated from the spleen using a naive CD4+ T cell isolation kit, which involved binding non-naive CD4+ T cells with a mixture of biotin-conjugated antibodies and anti-biotin beads. Highly pure naive CD4+ T cells were obtained by removing magnetically labeled non-naive CD4+ T cells, resulting in a purity of >90% as confirmed by flow cytometry. The purified CD4+ T cells were then cultured in a 24-well plate with RPMI-1640 medium containing 10% FCS, and stimulated with plate-bound anti-CD3 (5mg/ml) and soluble anti-CD28 (2mg/ml), the naive CD4+ T cells were stained with CFSE (2μmol/ml). Th1 polarization conditions included IL-12 (20ng/ml) and anti-IL-4 (10μg/ml). After 6 days of culture, cells will collected and analyzed by flow cytometry.

### 2.6 scRNA-seq data analysis

The Seurat package was used to convert the data from the GSE168299 dataset into Seurat objects. The Harmony package merges and removes batch effects from each sample of scRNA-seq data, removes mitochondrial genes, and analyzes the top 2,000 hypervariable genes. Next, T-distributed Stochastic Neighbor Embedding (tSNE) was used to visualize the merged results, T cell subsets were extracted for analysis and annotation, utilize the monocle package to perform a pseudotime trajectory analysis of CD Tn and CD4 Teff in T cells, focusing on the expression trajectories of Havcr2 and Nfil3 throughout cell development. Additionally, employ the Nebulosa package to investigate the co-expression patterns of Cd4, Havcr2, and Nfil3 in T cells.

### 2.7 Statistics

Data are presented as mean ± SD. The Shapiro-Wilk test was conducted on all datasets. For data that did not follow a normal distribution, the Mann-Whitney test was employed to assess the differences between the two groups. Conversely, for normally distributed data, the Student’s t-test was utilized to evaluate the differences between the two groups. The correlation between variables was analyzed using Spearman’s correlation test. Comparisons between multiple groups were performed using one-way Annova. Data processing and plotting were performed using GraphPad Prism 9.5. A *p*-value < 0.05 was considered statistically significant.

## 3 Results

### 3.1 Single-cell sequencing of COPD mice reveals the trajectory of Nfil3/Havcr2 in the activation process of lung CD4+ T cells

**Figure 1:**
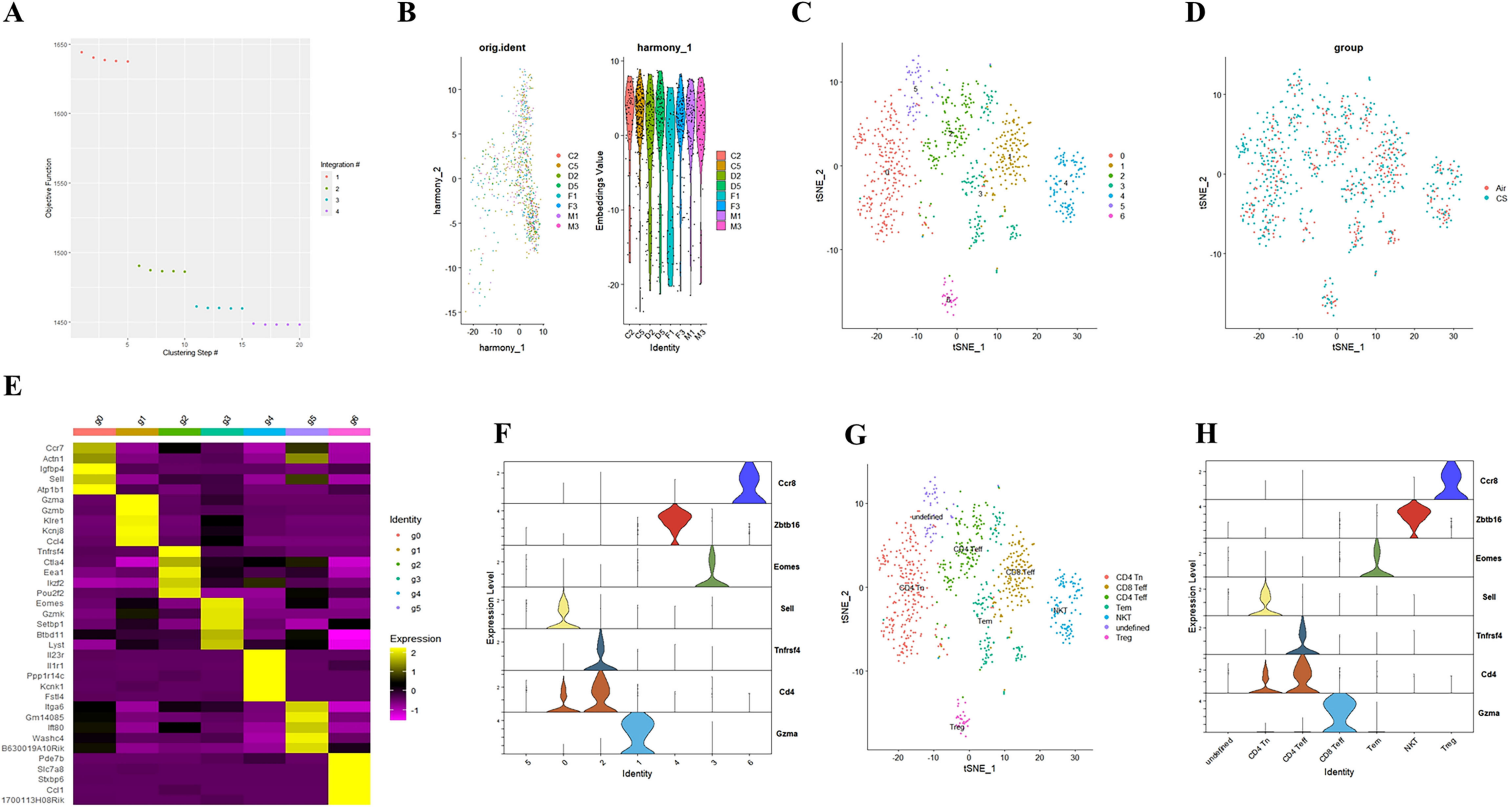
Integrating T cell single-cell sequencing data from lung tissues of mice exposed to air and those with COPD. **(A)** The Harmony algorithm is employed to integrate T cell data effectively. **(B)** Harmony algorithm calculates the characteristics of T cell data for each sample. **(C)** t-SNE analysis is performed on the single-cell sequencing data of T cells from the lung tissues of air and COPD mice. **(D)**. t-SNE analysis diagram is presented, illustrating the grouping of T cell single-cell sequencing data. **(E)** A differential gene expression heatmap is provided for T cell clusters (Log_2_FC). **(F)** A violin plot displays the marker genes for major T cell types in mouse lungs. **(G)**. The t-SNE analysis chart is annotated based on marker genes and differentially expressed genes. **(H)** A violin plot of the annotated T cell marker genes is also included.

To elucidate the potential role of NFIL3/Tim3 in regulating CD4+ T cell inflammation in the lungs of COPD patients, we first accessed the single-cell sequencing dataset GSE168299 from the GEO database (23), which includes C57BL mice used to model COPD through tobacco exposure. A total of 4 out of 6 mice (CS: C2, C5, D2, D5) and 4 air control mice (Air: F1, F3, M1, M3) were included in our analysis. We utilized the Seurat, Harmony, and Monocle packages in R version 4.3.1 for data reading and subsequent processing. After integrating the data, we identified a total of 13,328 cells, which were categorized into 26 cell clusters based on t-SNE cluster analysis (**Supplementary Figures 1A, B**). By calculating the differentially expressed genes for each cell cluster and identifying specific markers for certain cell types (**Supplementary Figures 1C, D**), we manually classified the 24 cell clusters into 12 distinct types: endothelial cells, myeloid cells, B cells, mast cells, T cells, fibroblasts, epithelial cells, NK cells, pericytes, SLC16A7+ cells, mesothelial cells, and red blood cells (**Supplementary Figure 1E**). T cells from both CS and Air mice were subsequently extracted for further analysis. We merged T cells from the 8 samples using the Harmony package (**Figures 1A, B**), successfully removing batch effects. A total of 804 T cells were clustered into 7 groups using the t-SNE algorithm (**Figures 1C, D**). Using the same methodology (**Figures 1E, F**), we manually classified the 7 T cell clusters into 7 types: CD4 Tn (naive CD4+ T cells), CD8 Teff (effector CD8+ T cells), Tem (central memory T cells), CD4 Teff (effector CD4+ T cells), Treg (regulatory T cells), NKT (natural killer T cells), and an undefined T cell subpopulation (**Figure 1G**). We observed that only CD4 Tn and CD4 Teff clearly expressed CD4 (**Figure 1H**), prompting us to focus our study on these two subtypes. To visualize gene expression, we employed the Nebulosa package to create gene expression density maps. By co-localizing the expression of Cd4 with key Th1 genes, Ifng and Tbx21, we ultimately determined the positioning of Th1 within the CD4 Teff population (**Supplementary Figures 2A–C**).

**Figure 2:**
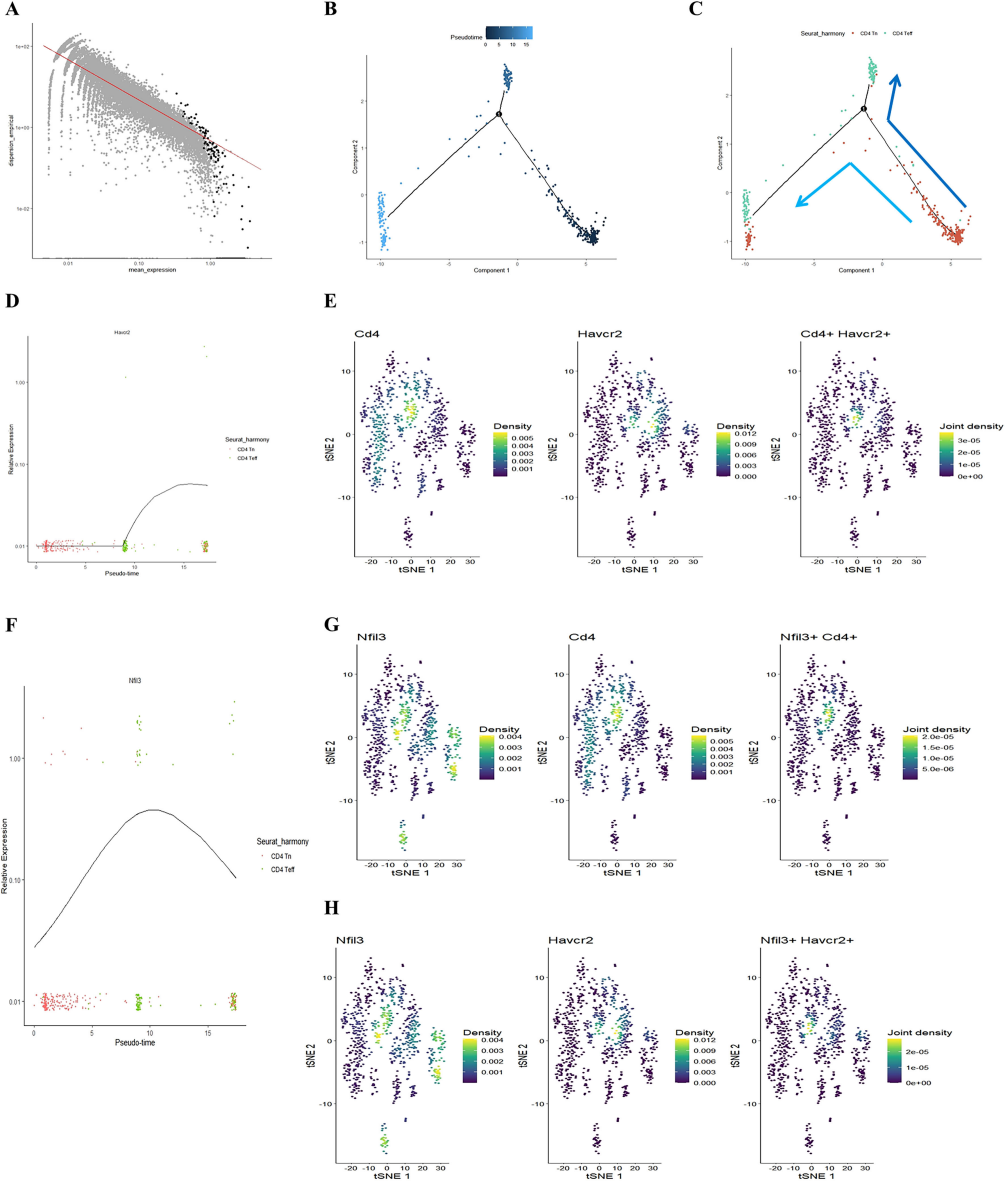
Illustrating the developmental trajectories of CD4 Tn and CD4 Teff cells, alongside the expression trajectory of Havcr2 and single-cell sequencing analysis indicates the predominant expression of Havcr2 and Nfil3 in Th1 cells. **(A–C)** present a pseudo-chronological analysis of CD4 Tn and CD4 Teff cells in both air-exposed and COPD mouse models. **(D)** illustrating the expression trajectory of Havcr2 during the development of CD4 Tn and CD4 Teff cells. **(E)** providing a co-expression analysis of Cd4 and Havcr2 in T cells.Single-cell sequencing analysis indicates the predominant expression of Havcr2 and Nfil3 in Th1 cells. **(F)** The expression trajectory of Nfil3 during the development of CD4 Tn and CD4 Teff cells. **(G)** Co-expression analysis of Nfil3 and Cd4 in T cells. **(H)** Co-expression analysis of Nfil3 and Havcr2 in T cells.

Our previous study found that initial CD4+ T cells (CD4+ Tn) were overactivated into effector memory CD4+ T cells (CD4+ Tem) in the lungs of COPD mice (24). Building on this, we employed the Monocle package to analyze the quasi-chronological developmental trajectories of CD4 Tn and CD4 Teff in single-cell data (**Figures 2A–C**). Our findings indicated that CD4+ T cells in mouse lung tissue predominantly followed the developmental trajectory from CD4 Tn to CD4 Teff. Following one cell fate selection, CD4 Tn primarily differentiated into CD4 Teff. Notably, the expression of Havcr2 gradually increased, peaking during the differentiation process from CD4 Tn to CD4 Teff (**Figure 2D**), while the expression of Nfil3 preceded that of Havcr2, reaching its peak upon the emergence of CD4 Teff (**Figure 2F**). Concurrently, we analyzed the co-expression of Cd4, Havcr2, and Nfil3 in T cell subsets (**Figures 2E, G, H**). Cd4, Havcr2, and Nfil3 were predominantly co-expressed at the Th1 position in CD4 Teff, suggesting that Nfil3 and Havcr2 may play a role in the activation and Th1 polarization of CD4+ T cells.

### 3.2 Tobacco exposure and Th1 polarization upregulate the expression of Tim3 and NFIL3 in mouse CD4+ T cells

**Figure 3:**
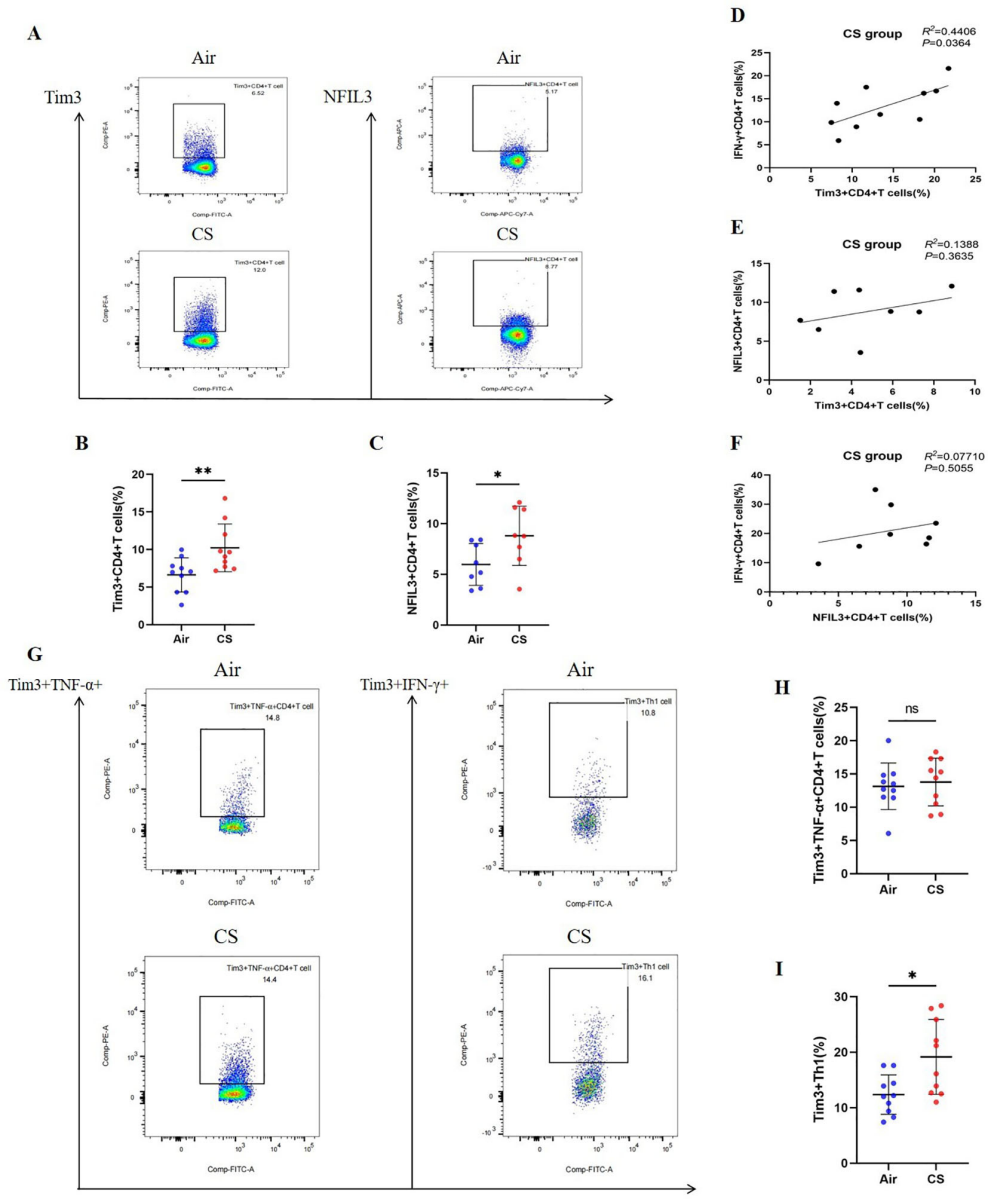
Exposure to tobacco enhances the expression of Tim3 and NFIL3 in CD4+ T cells within the lungs of mice and tobacco exposure induces the up-regulation of Tim3 in IFN-γ+CD4+T cells within the mouse lung, while Tim3+TNF-α+CD4+T cells do not show a significant impact. **(A)** Flow cytometry plots illustrate the expression levels of Tim3 and NFIL3 in lung CD4+ T cells of mice exposed to air versus those exposed to tobacco. **(B)** The frequency of Tim3+ CD4+ T cells in lung tissue is compared between mice in the Air group and CS group (n=10). **(C)** The frequency of NFIL3+ CD4+ T cells in lung tissue is compared between mice in the Air group and CS group (n=8). **(D–F)** Spearman’s correlation analysis is performed on IFN-γ+ CD4+ T cells, NFIL3+ CD4+ T cells, and Tim3+ CD4+ T cells in the CS group. **(G)** Representative flow cytometry images depict the expression of Tim3 on TNF-α+CD4+T cells and IFN-γ+CD4+T cells in the lungs of mice exposed to air and tobacco. **(H, I)** Comparisons between Tim3+TNF-α+CD4+T cells and Tim3+IFN-γ+CD4+T cells in lung tissue of mice from the Air group and CS group (n=10). The data, representative of three independent experiments, are presented as means ± SD. Group differences are assessed using a non-parametric test (Student’s t-test). * *P* < 0.05.

We followed the research group’s previous modeling method (21) to construct Chronic Obstructive Pulmonary Disease (COPD) and control mice (**Supplementary Figure 3A**). Flow cytometry was employed to detect relevant indicators of CD4+ T lymphocytes in mice from the tobacco exposure group (CS) and the air group (Air) (**Supplementary Figures 4**–**6**). Additionally, hematoxylin and eosin (HE) staining was utilized to assess lung tissue pathology. Consistent with earlier studies (26, 27), the lung tissue of COPD mice exhibited notable alveolar space expansion and an increased alveolar lining distance (Lm) (**Supplementary Figure 7**). Furthermore, lung CD4+ T cells, including IFN-γ+CD4+ T cells (Th1) and CD4+ Tem cells, were significantly elevated, while the expression of TNF-α was also increased. In contrast, CD4+ Tn cells were significantly reduced (**Supplementary Figures 3B–H**). We also compared the expression levels of Tim3 and NFIL3 in lung CD4+ T cells between the Air and CS groups. Chronic tobacco exposure significantly enhanced the expression of Tim3 and NFIL3 in CD4+ T cells (**Figures 3A–C**), suggesting that these markers may be associated with alterations in lung CD4+ T cells induced by tobacco exposure. Subsequently, we investigated the correlation between IFN-γ, Tim3, and NFIL3 in CD4+ T cells of tobacco-exposed mice. Our findings revealed a significant positive correlation between the percentage of Tim3+ CD4+ T cells and Th1 in the lungs of tobacco-exposed mice (**Figure 3D**). However, we did not observe detectable differences in the percentages of Tim3+ CD4+ T cells and NFIL3+ CD4+ T cells, nor in the percentages of IFN-γ+ CD4+ T cells and NFIL3+ CD4+ T cells in the lungs of tobacco-exposed mice (**Figures 3E, F**).

**Figure 4:**
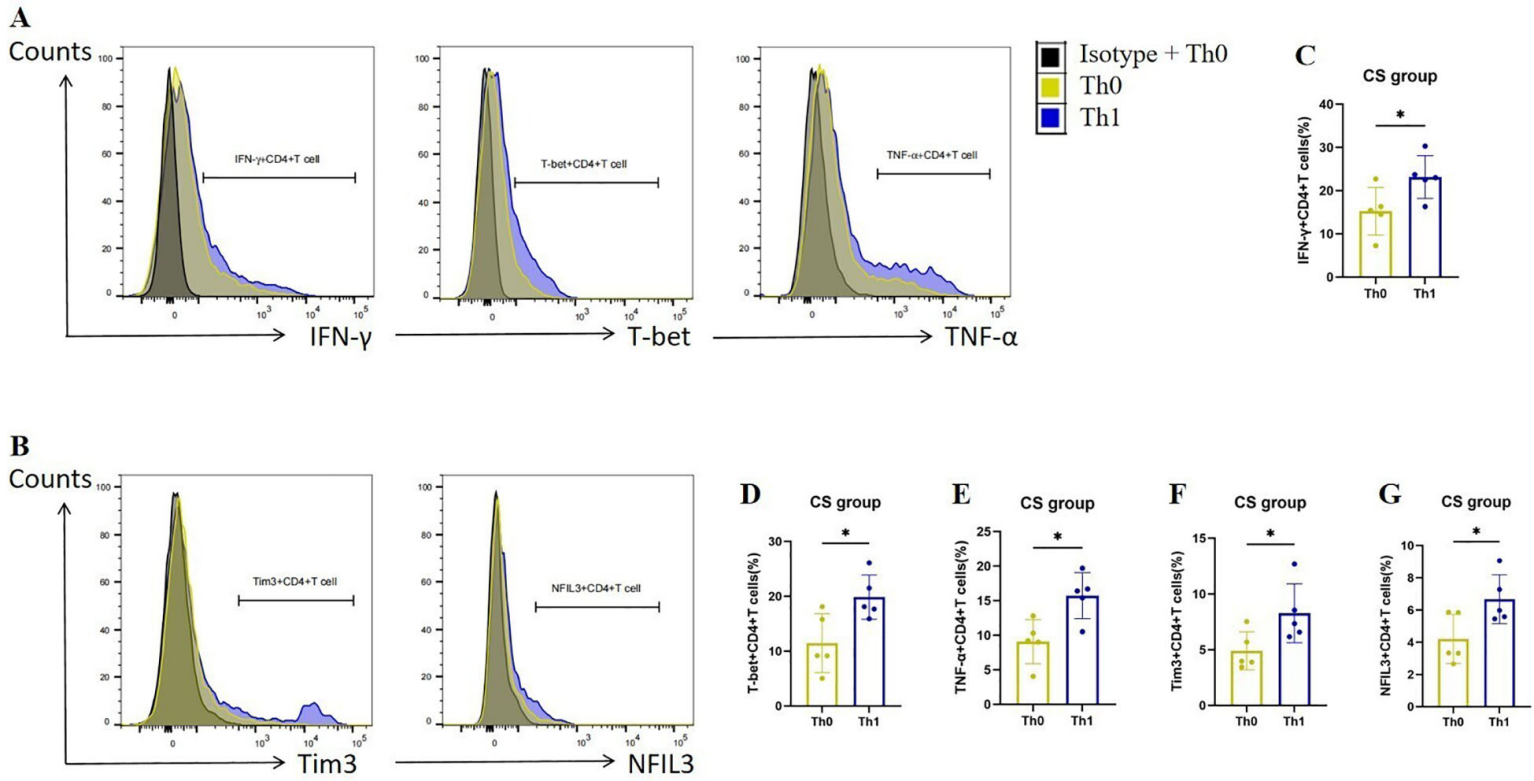
Th1 polarization enhances the expression of Tim3 and NFIL3 in CD4+ T cells following tobacco exposure. **(A)** Representative flow cytometry data illustrating the expression of IFN-γ, T-bet, and TNF-α in initial CD4+ T cells from the CS group under Th0/Th1 polarization conditions. **(B)** Representative flow chart depicting the expression of Tim3 and NFIL3 in initial CD4+ T cells from the CS group under Th0/Th1 polarization conditions. **(C–G)** Comparison of the expression frequencies of IFN-γ, T-bet, TNF-α, Tim3, and NFIL3 in initial CD4+ T cells from the CS group under Th0/Th1 polarization conditions (n=5). Data are representative of three independent experiments and are presented as medians ± SD. Differences between groups were analyzed using a non-parametric test (Student’s t-test).

To verify the up-regulation of Tim3 and NFIL3 expression during Th1 activation in COPD mice, we collected spleen CD4+ Tn cells from mice in the CS group, cultured them under Th0 and Th1 polarizing conditions, and assessed relevant indicators. Consistent with previous studies, CD4+ T cells exhibited a marked increase in the expression of IFN-γ, T-bet, and TNF-α following Th1 polarization (**Figures 4A, C–E**). Additionally, we observed that CD4+ T cells exhibited significantly higher expression of Tim3 and NFIL3 during Th1 polarization after oxidation (**Figures 4B, F, G**). This finding aligns with our single-cell sequencing results, which indicated that Havcr2 and Nfil3 are progressively up-regulated during the activation of CD4+ T cells in COPD mice.

### 3.3 Tobacco exposure stimulates the expression of Tim3+Th1 in mouse lungs

To further clarify the expression characteristics of Tim3 in lung CD4+ T cells stimulated by tobacco, we analyzed Tim3 expression in IFN-γ+ CD4+ T cells and TNF-α+ CD4+ T cells. As shown in **Figures 3G–I**, there is no significant difference in Tim3 expression in TNF-α+ CD4+ T cells between the Air group and the CS group. However, the Tim3+ IFN-γ+ CD4+ T cells in the CS group are significantly elevated compared to those in the Air group. Additionally, we examined Tim3 expression in CD4+ Tcm, CD4+ Tem, and CD4+ Tn. Although we did not observe marked differences in Tim3 expression among these three types of CD4+ T cells (**Supplementary Figure 8**), we did find that Tim3+ CD4+ Tem levels are higher in COPD mice than in control mice. These results indicate that chronic tobacco stimulation primarily leads to increased Tim3 expression in effector CD4+ T cells, particularly Th1.

**Figure 5:**
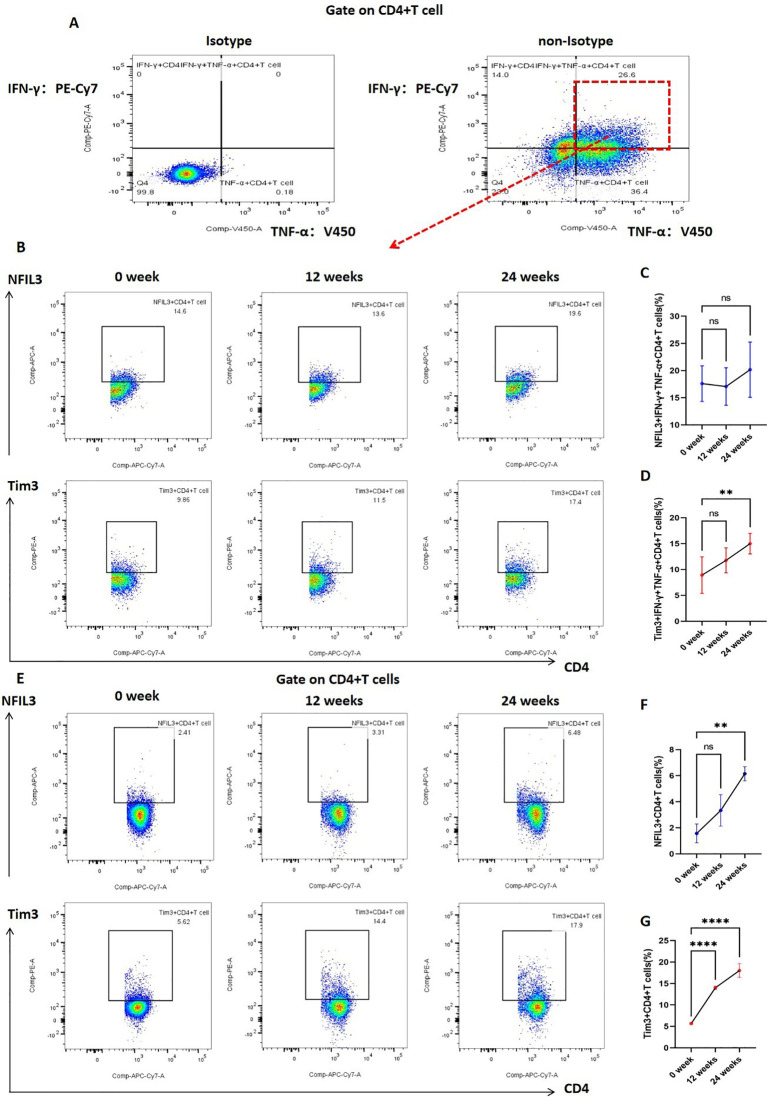
Expression of NFIL3 and Tim3 in lung CD4+T cells and IFN-γ+TNF-α+CD4+T cells of mice exposed to tobacco for 0, 12, and 24 weeks. **(A)** Gating strategy for IFN-γ+TNF-α+CD4+ T cells. **(B–D)** Typical flow cytometry and comparative analysis of NFIL3 and Tim3 expression in lung IFN-γ+TNF-α+CD4+T cells of mice exposed to tobacco for 0, 12, and 24 weeks (n=3). **(E–G)** Typical flow cytometry and comparative analysis of NFIL3 and Tim3 expression in mouse lung CD4+ T cells at 0, 12, and 24 weeks of tobacco exposure (n=3). The data are presented as means ± SD and differences between groups were analyzed using one-way Annova test. ** *P* < 0.01, **** *P* < 0.0001.

In addition, we investigated the expression of Tim3 and NFIL3 in CD4+ T cells and IFN-γ+TNF-α+CD4+ T cells at various time points (0, 12, and 24 weeks) during tobacco exposure. As illustrated in **Figure 5**, there was no significant difference in the expression of NFIL3 and Tim3 in IFN-γ+TNF-α+CD4+ T cells at 0 and 12 weeks post-tobacco exposure. However, at 24 weeks, Tim3 expression was significantly elevated in IFN-γ+TNF-α+CD4+ T cells, whereas NFIL3 did not show a significant increase (**Figures 5A–D**). Building on these findings, we further analyzed the expression of NFIL3 and Tim3 in CD4+ T cells across the three time points. Notably, Tim3 was significantly up-regulated in CD4+ T cells by 12 weeks following tobacco exposure, while the increase in NFIL3 at this time point was not significant. It was only at 24 weeks post-exposure that NFIL3 exhibited a relatively significant increase (**Figures 5E–G**). These results indicate that the sensitivity of Tim3 in CD4+ T cells to tobacco stimulation is markedly higher than that of NFIL3, as evidenced by the early and pronounced increase in expression levels.

### 3.4 Tim3 inhibits tobacco exposure-promoted emphysema formation, CD4+ T cell activation and IFN-γ expression in mice

**Figure 6:**
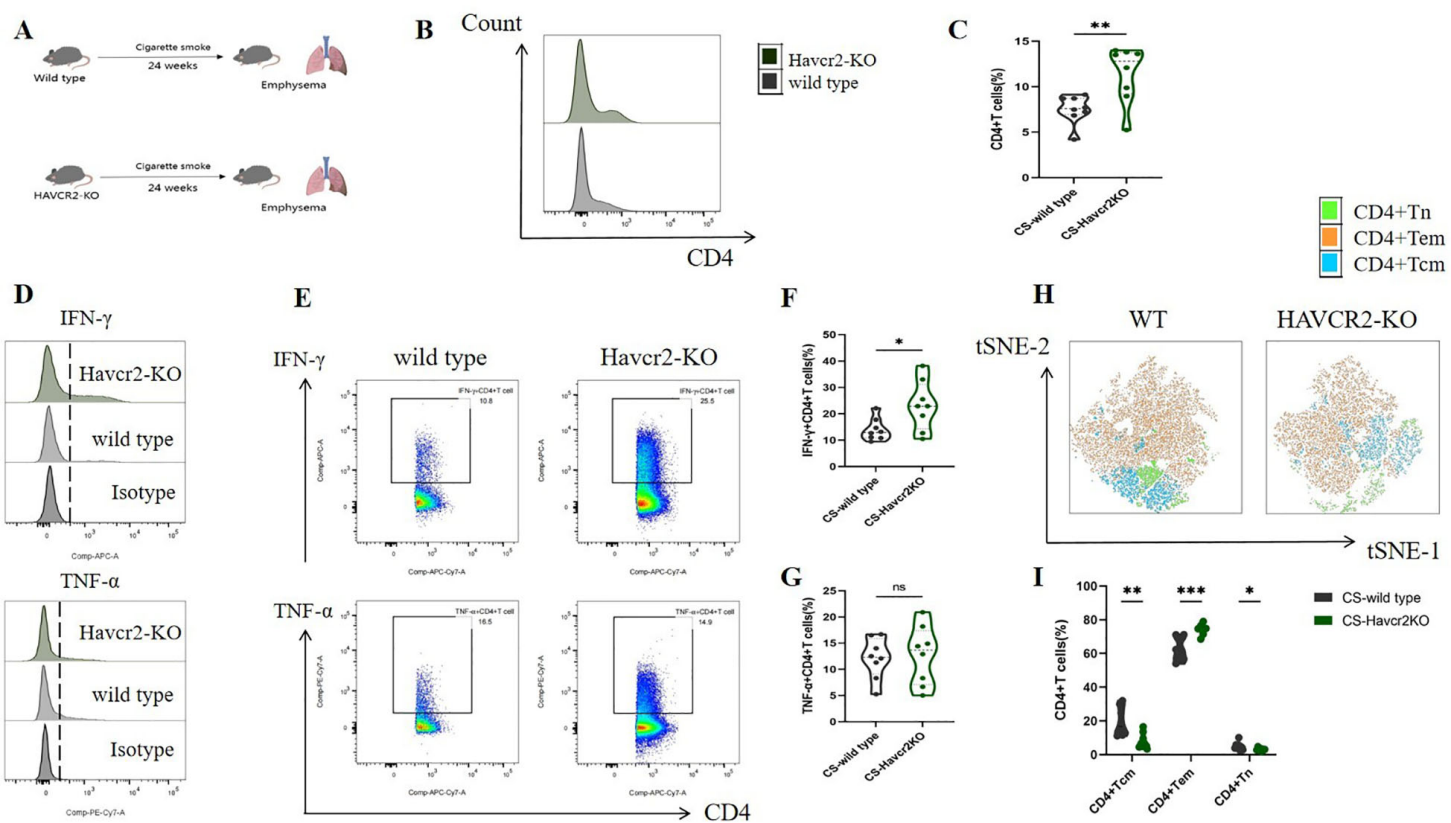
Knocking out Havcr2 further enhances tobacco exposure-induced CD4+T cell infiltration in mouse lungs, leading to increased expression of IFN-γ and CD4+Tem in lung CD4+T cells, while inhibiting the expression of CD4+Tcm and CD4+Tn. The expression of TNF-α remained unaffected. **(A)** A schematic diagram illustrating COPD modeling in wild-type and Havcr2-KO mice. **(B–G)** Comparisons of IFN-γ, and TNF-α levels in CD4+T cells between the WT and Havcr2-KO COPD mice. **(H, I)** Flow cytometric tSNE plots were used to compare lung CD4+Tn, CD4+Tcm, and CD4+Tem cells in WT and Havcr2-KO COPD mice. The data, based on three independent experiments, are presented as means ± SD and differences between groups were analyzed using a non-parametric test (Student’s t test). * *P* < 0.05, **** *P* < 0.0001.

Through bioinformatics analysis and animal experiments, we elucidated the correlation between Tim3 and IFN-γ in CD4+ T cells under tobacco exposure. However, direct evidence supporting the notion that Tim3 regulates the pro-inflammatory transformation of CD4+ T cells remains lacking. To further investigate the regulatory role of Tim3 in CD4+ T cell immune inflammation induced by tobacco smoke, we generated Havcr2 knockout mice (Havcr2-KO, **Supplementary Figures 9**, **10**) and subjected them to continuous tobacco exposure for 24 weeks (**Figure 6A**). Subsequently, we assessed the expression of CD4+ T cells in the lungs of both Havcr2-KO and tobacco-exposed mice concurrently. Our findings revealed that the knockout of Havcr2 resulted in a further increase in CD4+ T cell expression in the lungs (**Figures 6B, C**), indicating that Tim3 functions as an inhibitory factor for CD4 expression and that the absence of Havcr2 exacerbated lung disease associated with tobacco exposure.

We also measured the expression levels of IFN-γ and TNF-α in lung CD4+ T cells from both Havcr2-KO and wild-type (WT) mice, as well as the activation status of CD4+ T cells. In comparison to wild-type mice, IFN-γ+ CD4+ T cells in the lungs of Havcr2-KO mice were significantly up-regulated, whereas no substantial difference was observed in TNF-α+ CD4+ T cells (**Figures 6D–G**). This suggests that Tim3 primarily targets IFN-γ to inhibit lung CD4+ T cell inflammation in tobacco-exposed mice. Notably, we observed a significant up-regulation of CD4+ Tem cells, while CD4+ Tn and central memory CD4+ T cells (CD4+ Tcm) were significantly down-regulated in Havcr2-KO mice (**Figures 6H, I**), indicating that the knockout of Havcr2 ultimately led to an infiltration of CD4+ T cells in the lungs that were predominantly CD4+ Tem cells, which exert effector functions.

**Figure 7:**
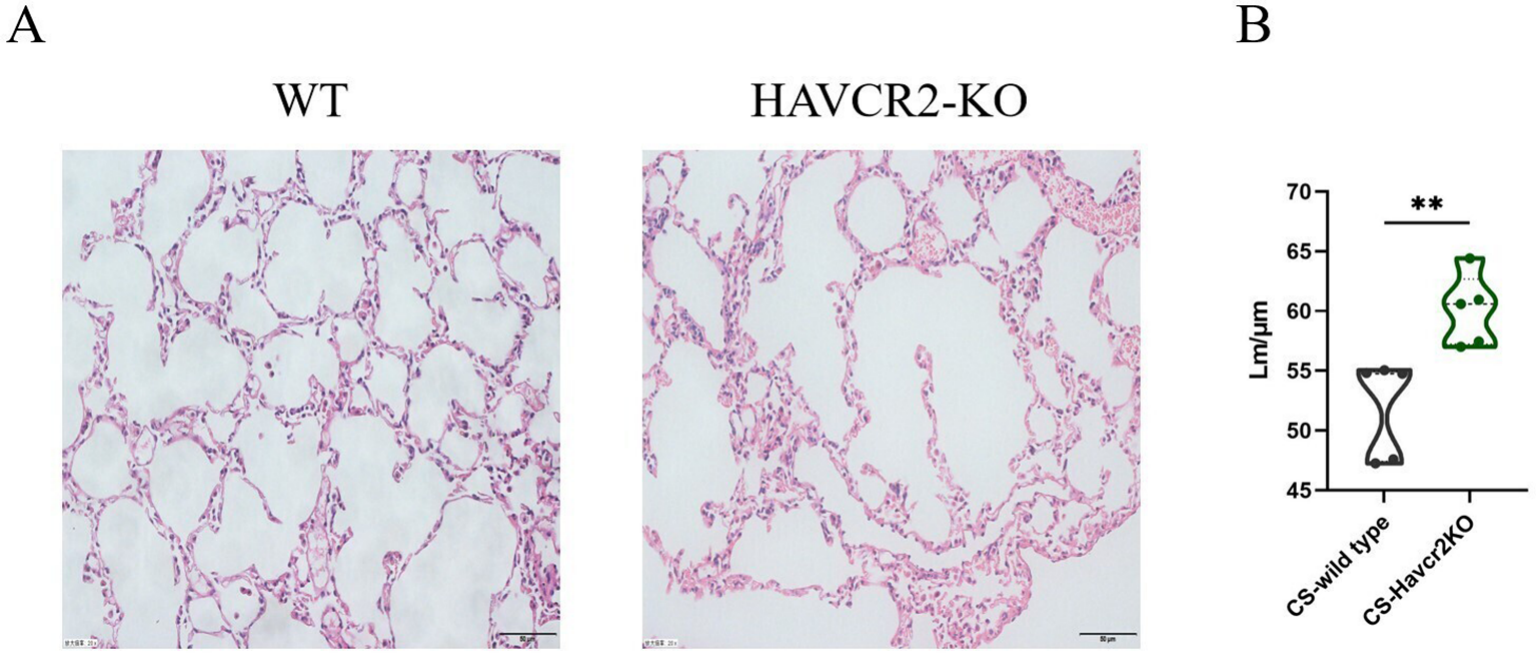
Knockout of Havcr2 exacerbates emphysema formation in C57BL/6J mice exposed to chronic tobacco. **(A)** Representative tissue images of lung H&E staining in WT COPD mice compared to Havcr2-KO COPD mice. **(B)** Lung Lm measurements show a significant difference between the two groups (n=5). data representing three independent experiments and presented as means ± SD. Group differences were analyzed using a non-parametric Student’s t test. ** *P* < 0.01.

In this study, we examined HE-stained sections of lung tissue from wild-type and Havcr2 knockout (KO) mice following chronic tobacco exposure to elucidate the role of Tim3 in emphysema formation. As illustrated in **Figure 7A**, after 24 weeks of chronic tobacco exposure, Havcr2-KO mice exhibited more pronounced alveolar destruction compared to wild-type mice. This observation was further validated by quantifying and comparing the mean linear intercept (Lm) between the two groups (**Figure 7B**). These findings suggest that Tim3 plays a pivotal role in the onset and progression of chronic obstructive pulmonary disease (COPD) in tobacco-exposed mice by influencing lung Th1 inflammation and the development of emphysema.

### 3.5 Knocking out Nfil3 promotes the activation of mouse lung CD4+T cells and the expression of IFN-γ, and inhibits the expression of Tim3

**Figure 8:**
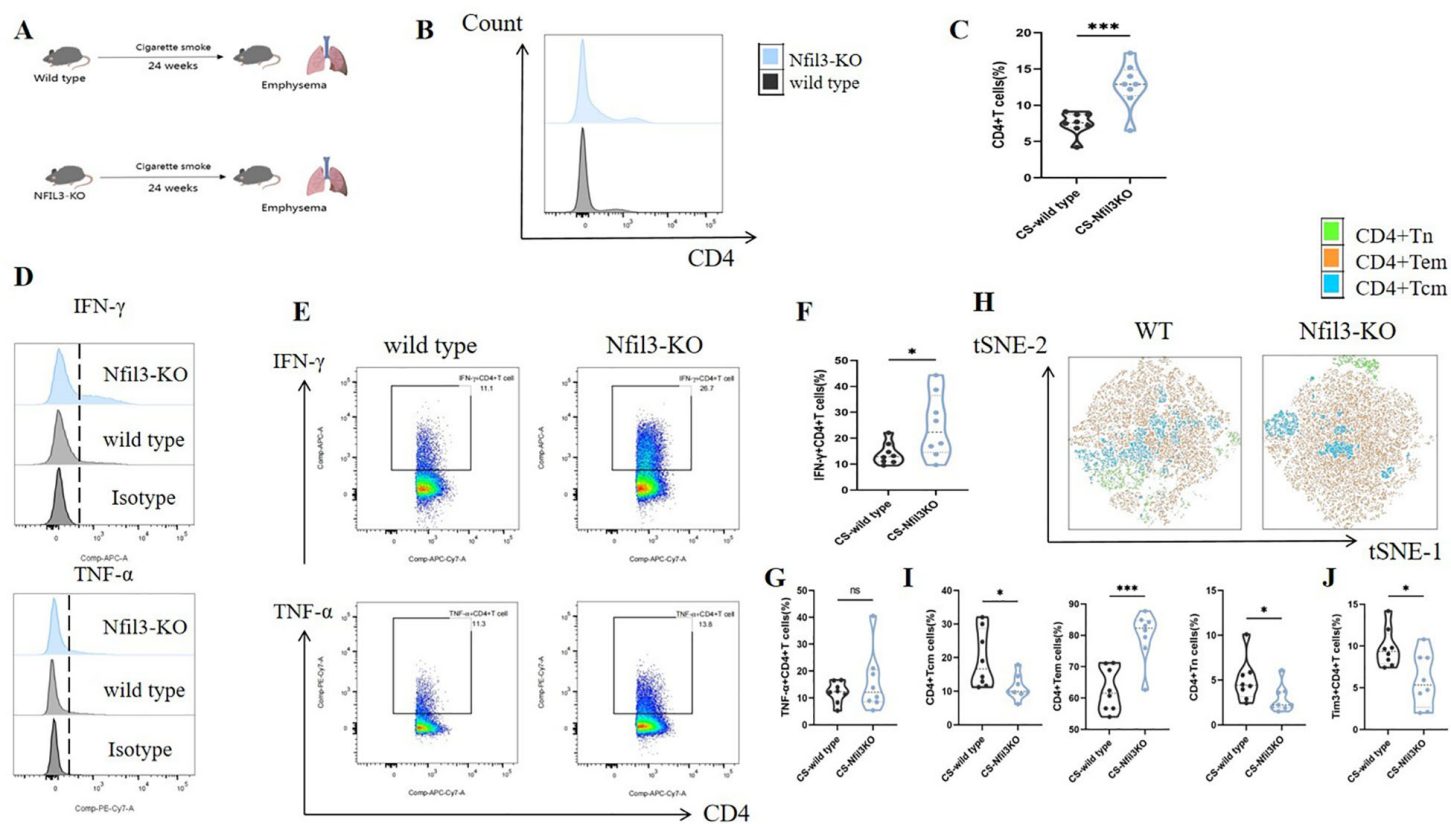
Knocking out Nfil3 further enhances the infiltration of CD4+ T cells in mouse lung exposed to tobacco, leading to increased expression of IFN-γ and CD4+Tem while inhibiting CD4+Tcm, CD4+Tn, and Tim3+CD4+T cells, the expression of TNF-α shows no significant impact. **(A)** A schematic diagram illustrating COPD modeling in wild-type and NFIL3-KO mice is provided. **(B, C)** Comparisons of CD4+ T cells in lung tissue between wild-type COPD mice and Nfil3-KO COPD mice (n=8). **(D–G)** Representative flow cytometry data and comparison of IFN-γ and TNF-α in lung CD4+ T cells of WT and Nfil3-KO COPD mice (n=8). **(H–J)** Representative flow cytometric tSNE plots and comparison of lung CD4+Tn, CD4+Tcm, and CD4+Tem cells in WT and Nfil3-KO COPD mice (n=8). **(I)** Comparisons of Tim3+CD4+T cells in the lungs of WT and Nfil3-KO COPD mice (n=8). Data from three independent experiments are shown as means ± SD. Group differences were analyzed using a non-parametric test (Student’s t test or Mann-Whitney test). * *P* < 0.05, ** *P* < 0.01, *** *P* < 0.001.

In our previous study, we found that NFIL3 was differentially expressed in lung CD4+ T cells of tobacco-exposed mice; however, our results did not establish a correlation between NFIL3, Tim3, and IFN-γ in CD4+ T cells. To clarify the regulatory function of NFIL3 on CD4+ T cells, we generated NFIL3 knockout mice (NFIL3-KO, **Supplementary Figures 9**, **10**) and exposed them to tobacco for 24 weeks (**Figure 8A**). Consistent with the results from Havcr2-KO mice, CD4+ T cells in the lungs of Nfil3-KO mice exhibited increased susceptibility to tobacco exposure (**Figures 8B, C**). The populations of IFN-γ+CD4+ T cells and CD4+Tem were significantly up-regulated in Nfil3-KO mice, while CD4+Tcm and CD4+Tn were significantly down-regulated. There was no significant difference in TNF-α+CD4+ T cells compared to wild-type mice (**Figures 8D–I**). These results indicate that NFIL3, similar to Tim3, is an important immune target for inhibiting CD4 expression, activation, and suppressing Th1 inflammation. Additionally, we assessed the expression of Tim3+CD4+ T cells in both wild-type and Nfil3-KO mice (**Supplementary Figure 11**). Following the knockout of Nfil3, the expression of Tim3 in CD4+ T cells was diminished (**Figure 8J**), suggesting that NFIL3, as an upstream regulatory factor, plays a role in the expression of Tim3 in lung CD4+ T cells after tobacco exposure. This finding further elucidates the observed increase in Nfil3 prior to Havcr2 in the quasi-chronological analysis. Interestingly, after further comparison of the relevant indicators between Nfil3-KO mice and Havcr2-KO mice during the same period (**Supplementary Figures 12**–**15**), we did not observe any significant differences between the two types of COPD mice.

## 4 Discussion

In this study, we found that the expression of Tim3 in CD4+ T cells of emphysema mice exerts an inhibitory effect on IFN-γ+ CD4+ T cells (Th1) within the lung microenvironment. Specifically, chronic tobacco exposure was shown to promote Tim3 expression in CD4+ T cells in the lungs of emphysema mice. The expression of Tim3 may represent a protective mechanism by the body against abnormal CD4+ T cell inflammation, primarily functioning to limit IFN-γ expression. Additionally, Tim3 expression is predominantly regulated by the upstream factor NFIL3. Together, these characteristics form a signaling pathway that modulates inflammation in COPD-Th1 cells.

Previous studies have established that COPD exhibits features akin to autoimmune diseases, characterized by an imbalance in adaptive immunity dominated by T cells (25). In our study, we also demonstrated that mice with emphysema induced by chronic tobacco exposure exhibited significantly higher CD4 expression in the lung microenvironment, alongside abnormal activation of CD4+ Tem cells, with Th1 cells showing a differentiation advantage. Notably, we are the first to report that Tim3 and NFIL3 are highly expressed in lung CD4+ T cells of COPD mice, with Tim3 also being highly expressed on Th1 cells in the CS group. Through correlation analysis, we observed a significant positive correlation between the proportion of Tim3+ CD4+ T cells and the proportion of Th1 cells.

Tim3 is a crucial immune checkpoint that mediates intracellular inhibitory signals in immune cells. By analyzing single-cell sequencing data from COPD mice, we found that Havcr2 is progressively upregulated as CD4+ Tn cells transition to CD4+ Teff cells, peaking at the terminal stage of activation. Song et al. (26) reported that Tim3 was highly expressed in depleted CD4+ Tcm cells in patients with acute pneumonia as determined by RNA-seq technology. Anderson et al. (27) highlighted that the co-expression of Tim3 and PD-1 serves as a significant marker for assessing T cell functional exhaustion. However, our study revealed that Tim3 was also highly expressed in Th1 cells with effector function within the lungs of COPD mice. Further analysis of Tim3 expression in CD4+ Tcm, CD4+ Tem, and CD4+ Tn cells, as well as TNF-α+ CD4+ T cells, showed no significant differences between the Air group and the CS group. Monney et al. (28) found that Tim3 is expressed during the late stages of Th1 polarization, and Tim3-mRNA exhibiting spatiotemporal characteristics of expression that transition from lymph nodes to peripheral tissues throughout the initial to peak stages of experimental autoimmune cerebrospinal meningitis. This indicates that Tim3 does not facilitate T cell activation and differentiation, rather, it participates in T cell migration and effector functions. Our experimental results further underscore the specificity of Tim3 expression in Th1 cells, and Havcr2, which peak during the terminal stages of T cell activation in COPD mice. Therefore, We speculate that under chronic tobacco exposure, Tim3 in CD4+ T cells primarily inhibits the expression of IFN-γ in effector CD4+ T cells. NFIL3 has been identified as an important immune regulatory factor, and its inhibition can lead to a deficiency in IL-10 expression, potentially resulting in more severe autoimmune diseases (29–31). Additionally, NFIL3 is considered an upstream signal regulating Tim3 expression in T cells (19). Our analysis of single-cell data indicated that Nfil3 exhibited a trend of initial increase followed by decrease with the activation of CD4+ T cells, peaking in CD4+ Teff cells, and its pattern of change preceded that of Havcr2. Moreover, Nfil3 and Havcr2 were predominantly co-expressed in CD4+ Teff cells. Flow cytometric analysis of lung tissue from COPD mice demonstrated that NFIL3 is highly expressed in CD4+ T cells.

To verify the results of single-cell sequencing, we sorted spleen CD4+ Tn cells from COPD mice and cultured them under Th0 and Th1 polarized conditions to simulate the activation process of CD4+ Tn cells into CD4+ Teff cells. Our findings indicated that CD4+ T cells significantly expressed IFN-γ, TNF-α, and T-bet under Th1 polarization conditions, with notable increases in the expression of Tim3 and NFIL3. This suggests that the activation of CD4+ T cells is associated with elevated expression of Tim3 and NFIL3. However, the specific roles of NFIL3 and Tim3 in the Th1 polarization process in COPD mice remain unclear. To elucidate these roles, we generated Havcr2-KO and Nfil3-KO mice and subjected them to tobacco smoke exposure. We observed that the expression of CD4 in the lung tissue of these mice was significantly elevated, accompanied by pronounced alveolar septal destruction. Notably, the expressions of CD4+ Tn and CD4+ Tcm were significantly decreased, while CD4+ Tem expression was markedly increased. Following the knockout of Havcr2, IFN-γ+ CD4+ T cells exhibited heightened sensitivity to tobacco stimulation, whereas TNF-α levels remained unchanged. These observations confirm that Tim3 primarily influences IFN-γ, and the knockout of Havcr2 differentially remodels effector CD4+ T cells (CD4+ Tem and IFN-γ+ CD4+ T cells) and functionally stable CD4+ T cells (CD4+ Tn and CD4+ Tcm), leading to increased inflammatory infiltration and destruction of alveolar structures. The expression of Tim3 in Th1 cells may serve a self-activating role and transmit inhibitory signals to limit excessive Th1 activation. CD4+ T cells from Nfil3-KO mice exhibited properties similar to those from Havcr2-KO mice. By comparing lung CD4+ T cells in wild-type and Nfil3-KO mice, we found that the expression of Tim3+ CD4+ T cells was significantly down-regulated in Nfil3-KO mice, suggesting that the effect of Nfil3 knockout on CD4+ T cells may occur through the downregulation of Tim3 expression.

Previous studies have reported that during the construction of a COPD mouse model through passive smoke exposure, mice developed observable emphysema by the 12th week (32). This indicates that mice exhibit certain pathological characteristics of COPD after 12 weeks of passive smoking. Consequently, we selected mice exposed to tobacco for 12 weeks to evaluate the expression of Tim3 and NFIL3 in CD4+ T cells during the early stages of emphysema. Our findings revealed that in CD4+ T cells, exposure to tobacco for 12 weeks resulted in a significant increase in Tim3, while a notable increase in NFIL3 was not observed until week 24. In IFN-γ+TNF-α+CD4+ T cells, which exhibit a more pronounced pro-inflammatory phenotype, neither Tim3 nor NFIL3 displayed significant changes at week 12. However, after 24 weeks of continuous exposure, only Tim3 showed a significant increase in double-positive CD4+ T cells. These results suggest that Tim3 in CD4+ T cells is more sensitive to the stimulation of continuous tobacco smoke. NFIL3 is known to regulate the expression of Tim3 in T cells, a process mediated by IL-27 (19). Our previous study also indicated that the concentration of IL-27 in the plasma of smoking-related COPD patients increased significantly (24). This suggests the potential involvement of the IL-27/NFIL3/Tim3 axis in COPD mice. However, Chihara et al. (33) reported that IL-27 can regulate the expression of Tim3 in CD4+ T cells through the synergistic effects of PRDM1 and c-MAF. These findings imply that multiple IL-27-mediated pathways may regulate Tim3 expression in CD4+ T cells. Therefore, our study observed that Tim3 expression is more sensitive than NFIL3 following tobacco exposure, potentially due to the diversity of Tim3 regulatory mechanisms. Future research will require additional experiments and data to validate this inference.

Based on this, we continued to compare CD4+ T cell activation and Th1 expression between Nfil3-KO mice and Havcr2-KO mice. However, no statistically significant differences were observed between the two groups. This suggests that NFIL3 and Tim3 exert similar regulatory effects in inhibiting lung CD4+ T cell activation and Th1 polarization in COPD mice. Given that NFIL3 is an upstream regulator of Tim3, we can infer that NFIL3 primarily regulates Tim3 to exert an inhibitory effect on CD4+ T cells in the context of COPD.

Nonetheless, this study has several limitations: 1. It does not assess the expression levels of NFIL3 in CD4+ T cells of Havcr2 knockout mice compared to wild-type mice; 2. It lacks lung tissue pathology analysis in Nfil3 knockout COPD mice; 3. It does not investigate the upstream regulatory mechanisms of the NFIL3/Tim3 axis; 4. In this study, we selected whole-body knockout mice. Given that Tim3 is also expressed in myeloid cells and plays a role in regulating cytokine secretion and cell activation (34, 35), we plan to further elucidate our findings by generating CD4-specific Havcr2 and Nfil3 knockout COPD mice in future research.

## 5 Conclusions

Overall, we propose that the expression of Tim3 in an inflammatory environment serves as a protective mechanism for CD4+ T lymphocytes, enabling them to counteract chronic tobacco stimulation and prevent excessive immune-inflammatory responses. The expression of Tim3 is regulated by NFIL3 in CD4+ T cells. Additionally, Th1 amplification induced by tobacco stimulation can be partially inhibited via NFIL3/Tim3 signaling. However, in the absence of intervention, this protective mechanism is insufficient to completely mitigate Th1 inflammation in the lungs resulting from persistent tobacco exposure. Furthermore, we have identified the promoting role of Tim3 in the development of emphysema, which enhances our understanding of the pathogenesis of chronic obstructive pulmonary disease (COPD). By elucidating the central role of Tim3 in the pathogenesis of COPD, we suggest that new anti-inflammatory treatments targeting Tim3 may be applicable for COPD management.

## Data Availability

The datasets presented in this study can be found in online repositories. The names of the repository/repositories and accession number(s) can be found in the article/**Supplementary Material**.
