# Supplementary material for: NFIL3/Tim3 axis regulates effector Th1 inflammation in COPD mice
Source: Front Immunol. 2024 Nov 1;15:1482213. doi: 10.3389/fimmu.2024.1482213 (PMC11563780; doi:10.3389/fimmu.2024.1482213)
Supplement: Supplementary file 1 [file DataSheet1.zip › Supplementary Materials/Supplementary Figures.docx]

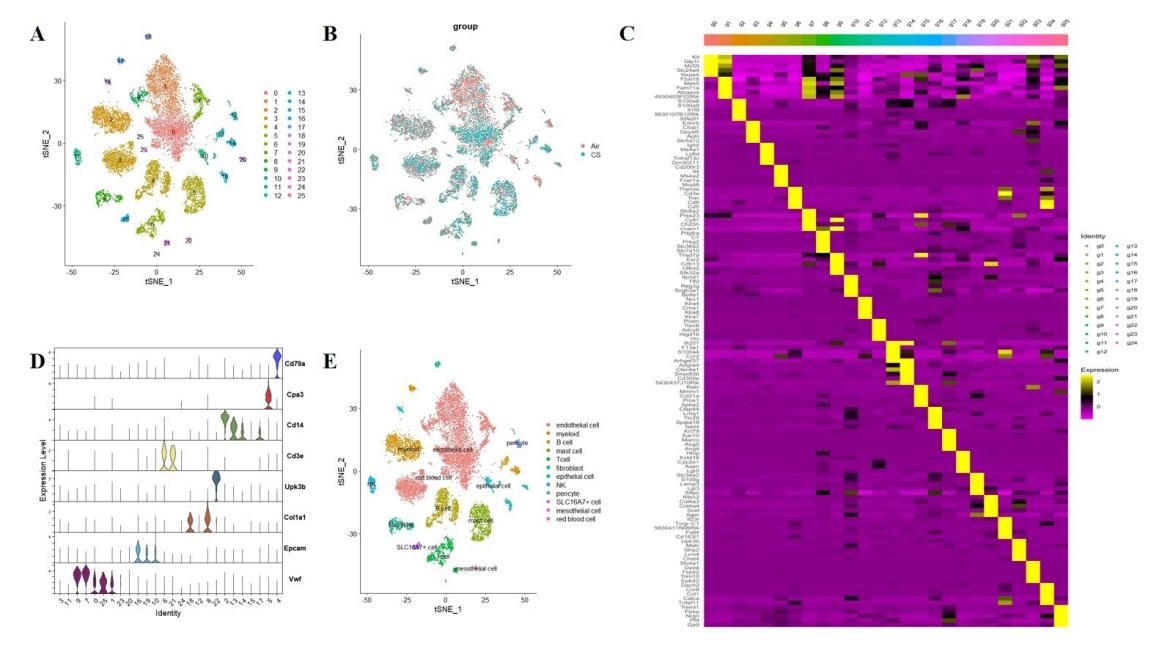


Supplementary Figure 1. Integrating single-cell sequencing data from lung tissues of mice exposed to air and those with Chronic Obstructive Pulmonary Disease (COPD). A. t-SNE analysis of single-cell sequencing data from the lung tissue of air-exposed and COPD mice. B. t-SNE analysis diagram grouped by single-cell sequencing. C. Heatmap illustrating differential gene expression across cell clusters (Log_2_FC). D. Violin plot depicting marker gene expression for major cell types in the mouse lung. E. Cell annotation of the t-SNE analysis chart based on marker genes and differentially expressed genes.


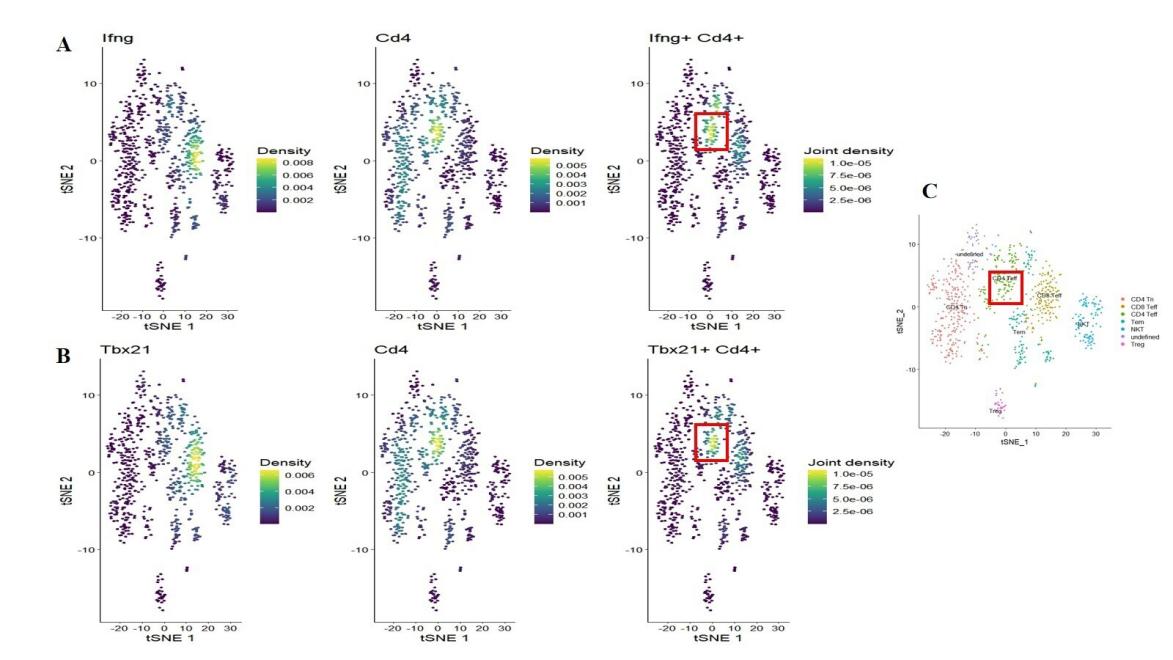


Supplementary Figure 2. Gene co-expression determining the specific position of Th1 cells in t-SNE analysis. A. Co-expression analysis of Ifng and Cd4 in T cells. B. Co-expression analysis of Tbx21 and Cd4 in T cells. C. Integration of the co-expression data for Ifng, Tbx21, and Cd4 is utilized to ascertain the specific position of Th1 cells within the single-cell sequencing data.


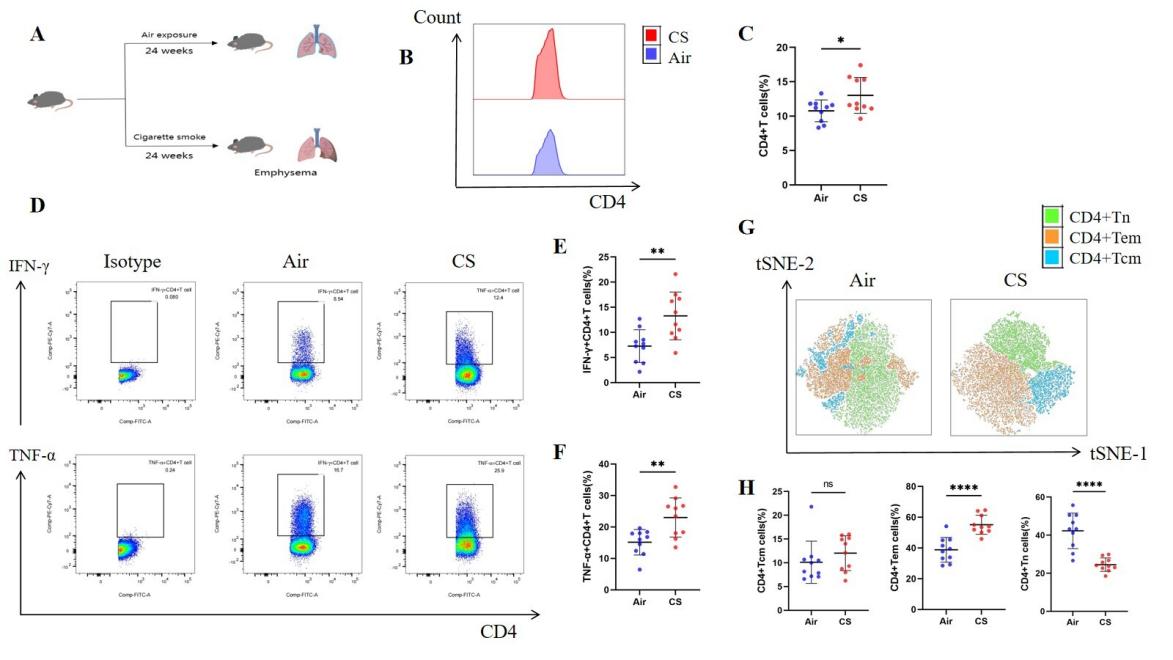


Supplementary Figure 3. Chronic exposure to tobacco enhances the presence of CD4+ T cells in the lungs of mice, leading to the activation of CD4+ T cells and increased expression of IFN-γ and TNF-α. A. Schematic diagram of COPD mouse model. B-C. Comparison of CD4+ T cells in lung tissue of air-exposed mice and tobacco-exposed mice (n=10). D-F. Representative flow cytometry and comparison of IFN-γ and TNF-α in lung CD4+T cells of mice in Air group and CS group (n=10). G-H. Representative flow cytometric tSNE diagrams and comparisons of lung central memory, effector memory, and naive CD4+ T cells in mice in the Air group and CS group (n=10). The data, representing three independent experiments, are displayed as means ± SD. Group differences were analyzed using a non-parametric test (Student's t test or Mann-Whitney test). * *P* < 0.05, ** *P* < 0.01, **** *P* < 0.0001.


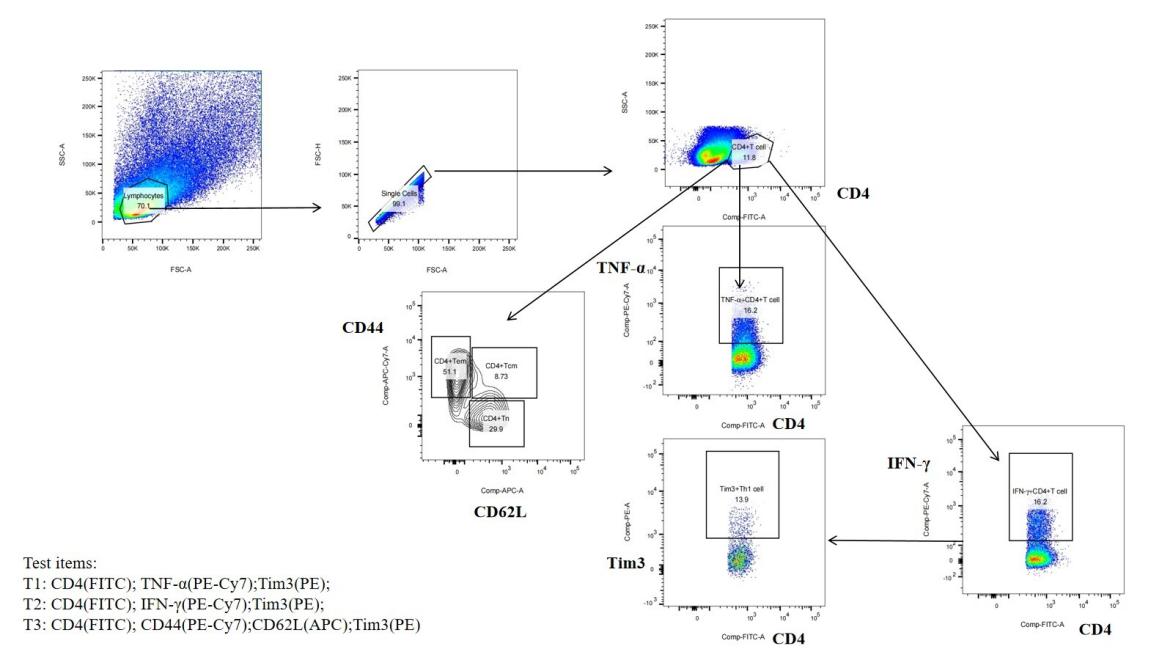


Supplementary Figure 4. Flow cytometry gating strategy for single-cell suspensions in lung tissue from wild-type air-exposed and cigarette smoke exposed mice (IFN-γ, TNF-α, Tim3+Th1, CD4+Tn, CD4+Tcm, CD4+Tem).


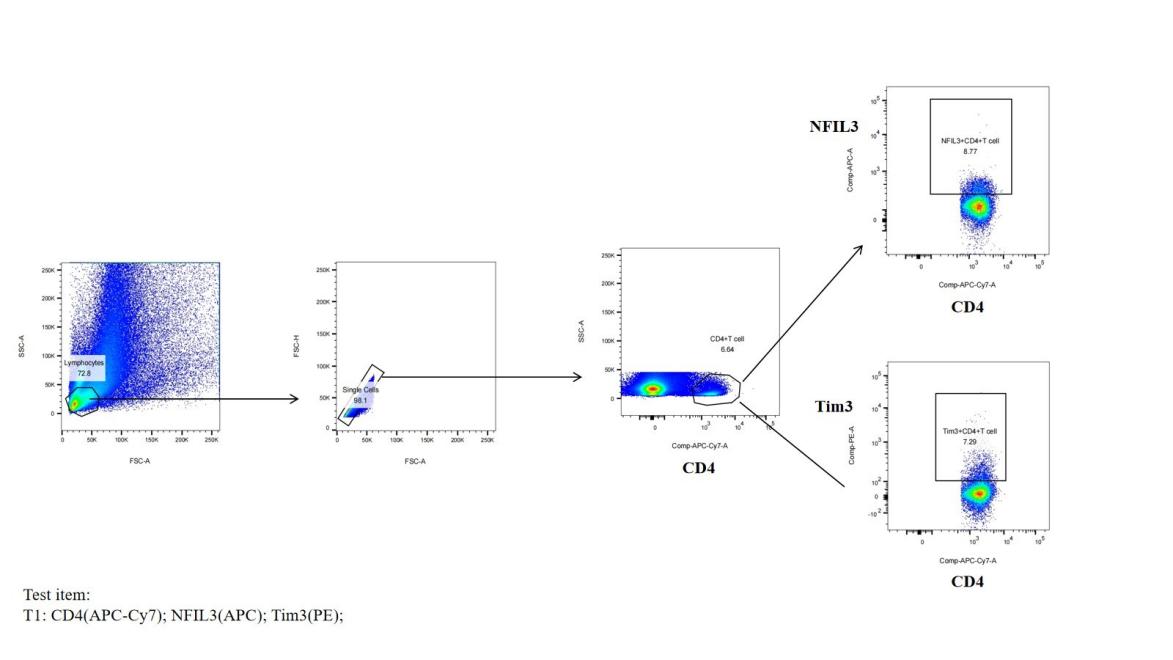


Supplementary Figure 5. Flow cytometry gating strategy for single-cell suspensions in lung tissue from wild-type air-exposed and cigarette smoke exposed mice (NFIL3, Tim3).


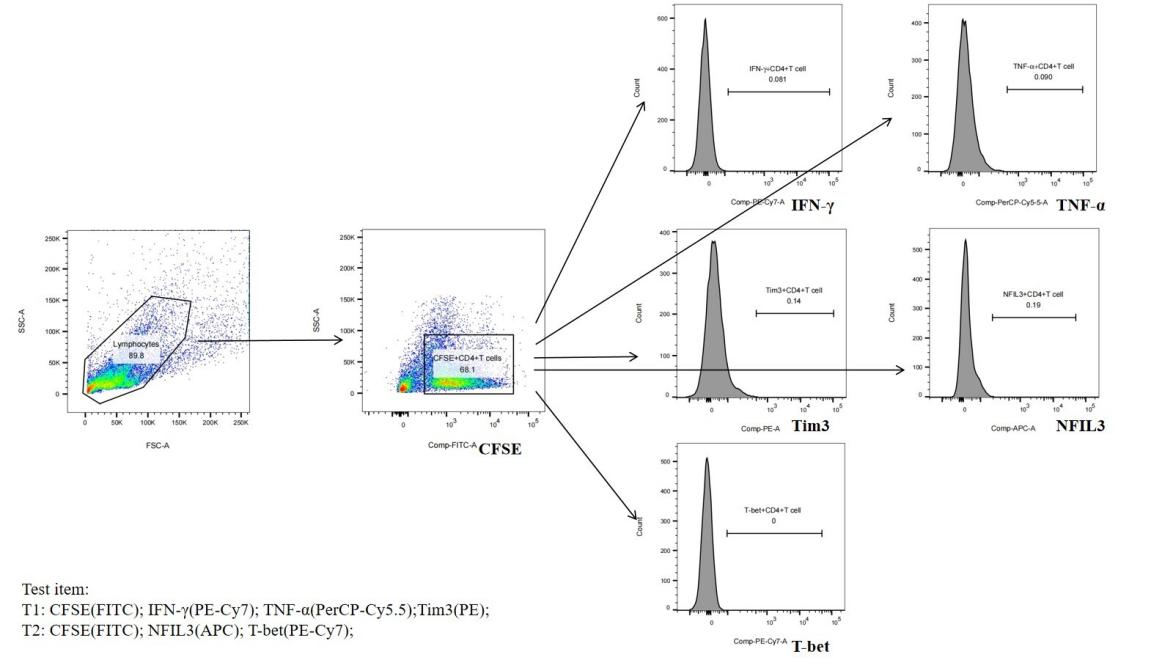


Supplementary Figure 6. Gating strategy after isolating naive CD4+ T cells from spleens of mice in Air group and CS group by flow cytometry (IFN-γ, TNF-α, Tim3, T-bet, NFIL3).


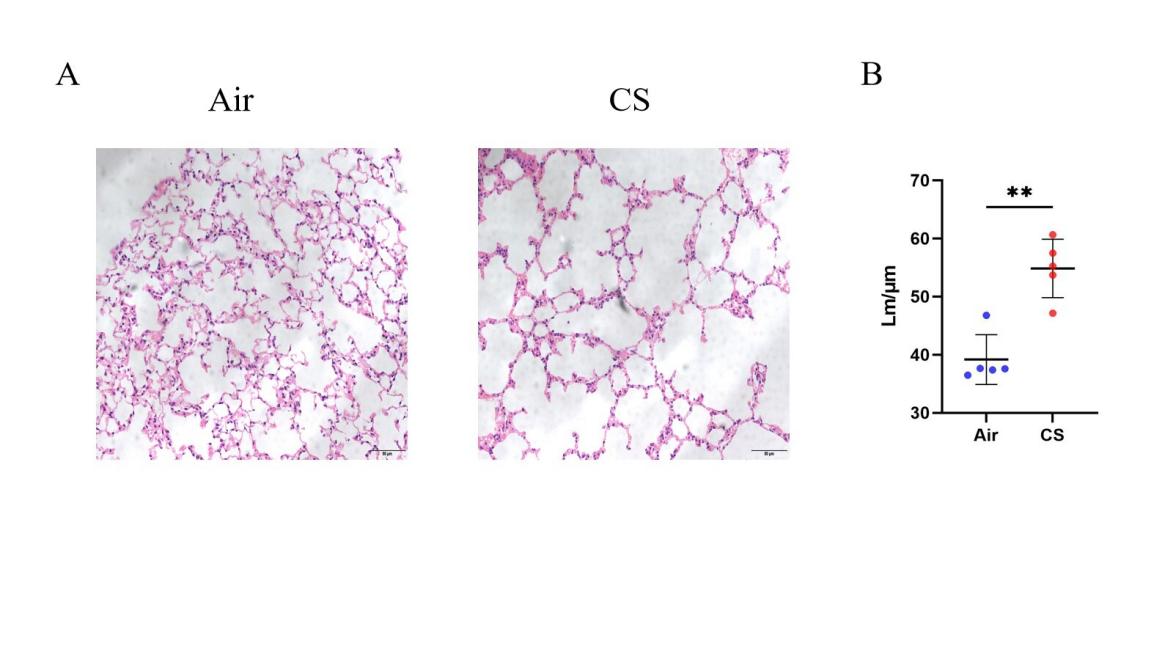


Supplementary Figure 7. Chronic exposure to tobacco leads to the development of emphysema in C57BL/6J mice. A. Representative tissue images show H&E staining of lungs from mice exposed to air and tobacco over the same time frame. B. Lung Lm comparison between air-exposed and tobacco-exposed mice (n=5) is presented. The data, representing three independent experiments, are displayed as means ± SD. Group differences were analyzed using a non-parametric test (Mann-Whitney test). *** means *P* value < 0.001.


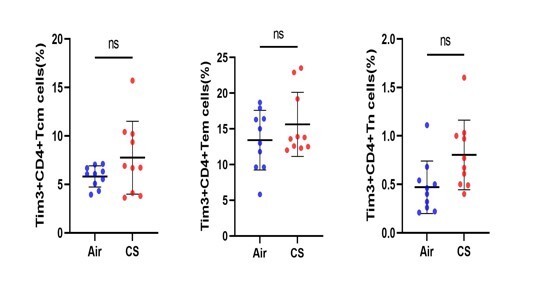


Supplementary Figure 8. Comparisons of Tim3 levels in lung CD4+Tcm, CD4+Tem and CD4+Tn cells in mice in the Air group and CS group (n=10). The data, representing three independent experiments, are displayed as means ± SD. Group differences were analyzed using a non-parametric test (Student's t test or Mann-Whitney test).


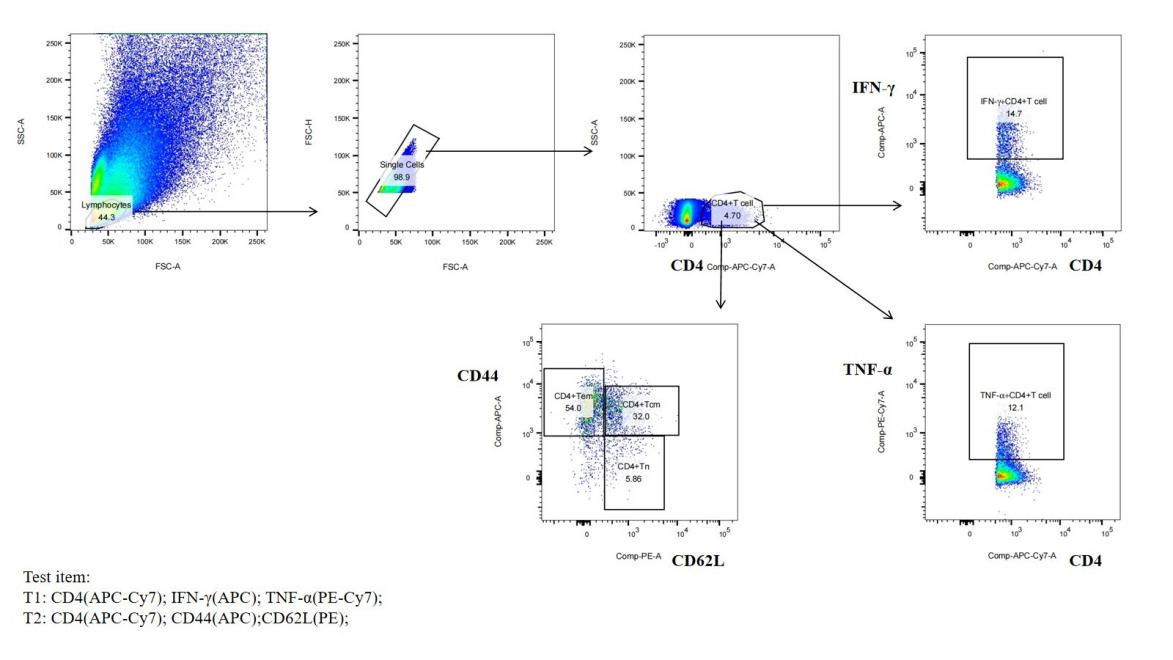


Supplementary Figure 9. Flow cytometry gating strategy for single cell suspensions in lung tissue from wild-type tobacco-exposed and HAVCR2/NFIL3-KO tobacco-exposed mice (IFN-γ, TNF-α, CD4+Tn, CD4+Tcm, CD4+Tem).


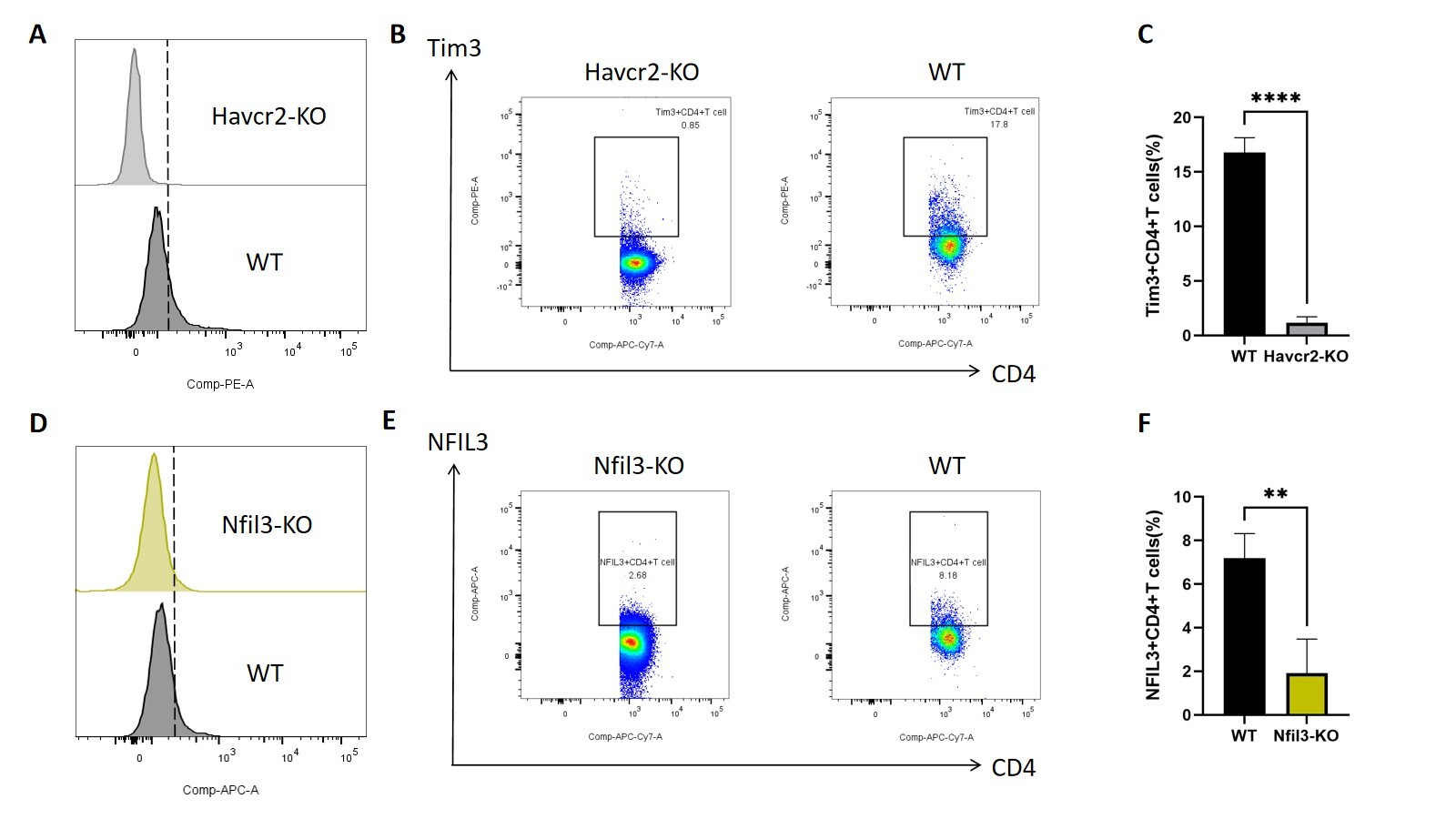


Supplementary Figure 10. The single-cell suspension of lung tissue of HAVCR2/NFIL3 knockout mice was labeled with Tim3 and NFIL3 by flow cytometry to verify the knockout efficiency. A-C. Typical flow cytometry and comparison of Tim3+CD4+T cell expression frequency between WT mice and Havcr2-KO mice (n=3). D-F. Typical flow cytometry and comparison of NFIL3+CD4+T cell expression frequency between WT mice and Nfil3-KO mice (n=3).


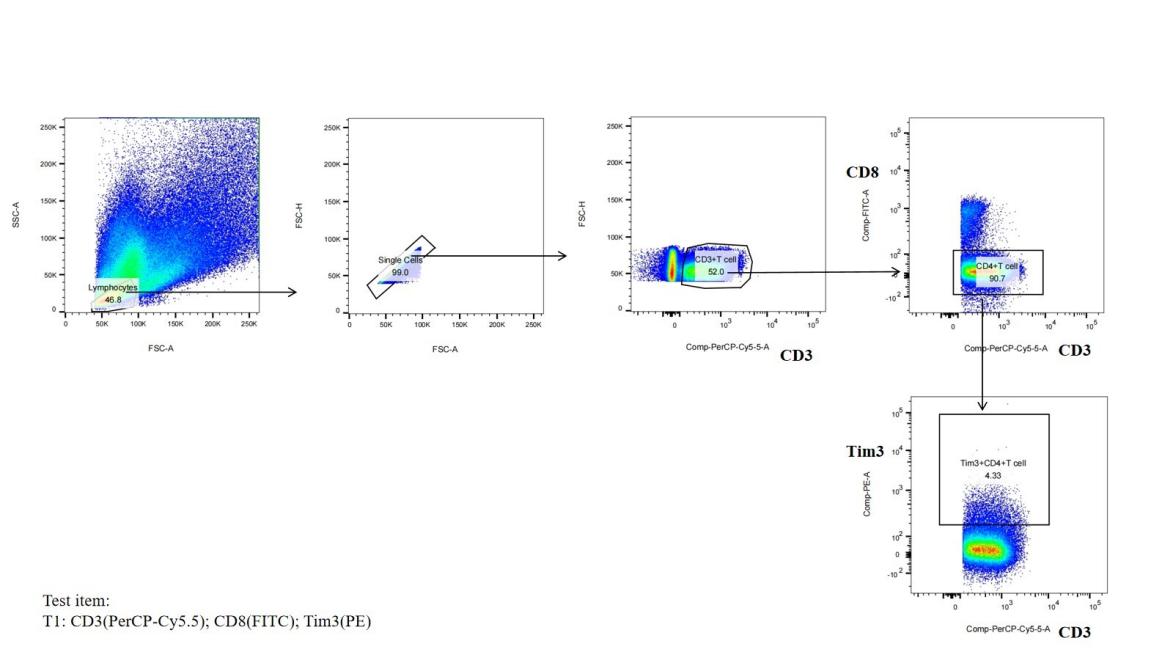


Supplementary Figure 11. Flow cytometry gating strategy for single-cell suspension in lung tissue of NFIL-KO mice in CS group (Tim3).


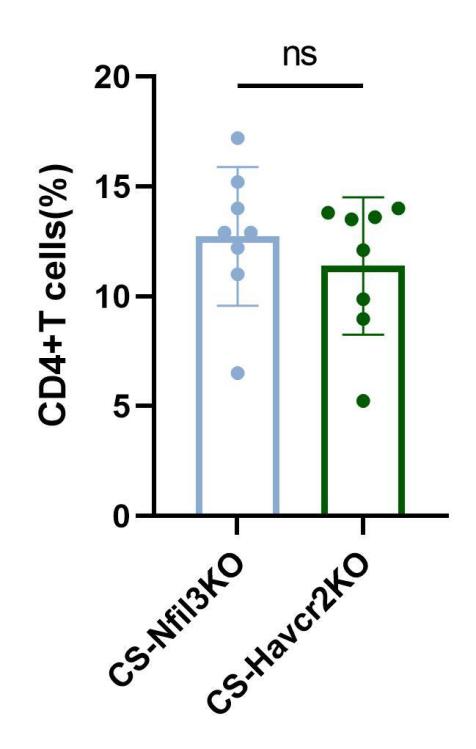


Supplementary Figure 12. Comparisons of CD4+T cells between the Nfil3-KO mice and Havcr2-KO COPD mice (n=8).The data, based on three independent experiments, are presented as means ± SD and differences between groups were analyzed using a non-parametric test (Student's t test).


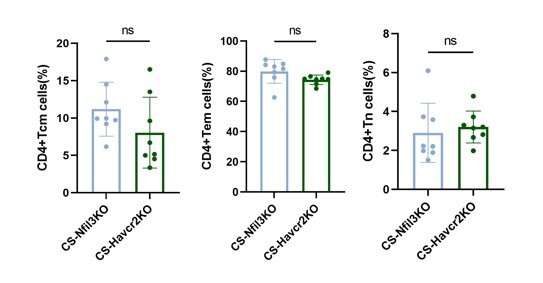


Supplementary Figure 13. Comparisons of CD4+Tcm, CD4+Tem, CD4+Tn cells between the Nfil3-KO mice and Havcr2-KO COPD mice (n=8).The data, based on three independent experiments, are presented as means ± SD and differences between groups were analyzed using a non-parametric test (Student's t test).


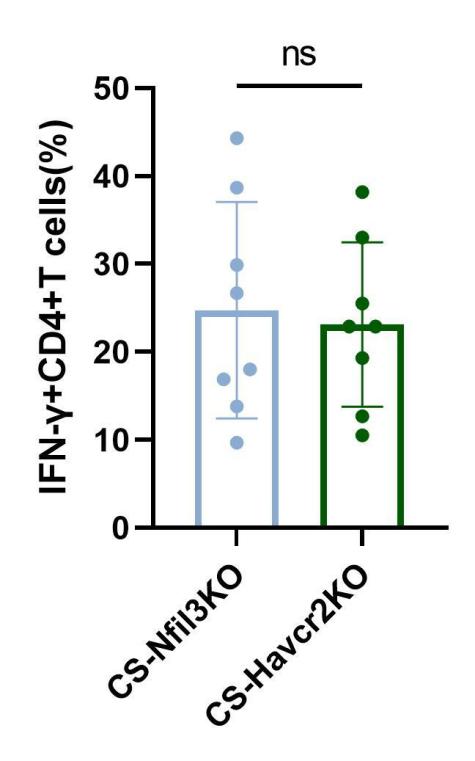


Supplementary Figure 14. Comparisons of IFN-γ in CD4+T cells between the Nfil3-KO mice and Havcr2-KO COPD mice (n=8).The data, based on three independent experiments, are presented as means ± SD and differences between groups were analyzed using a non-parametric test (Student's t test).


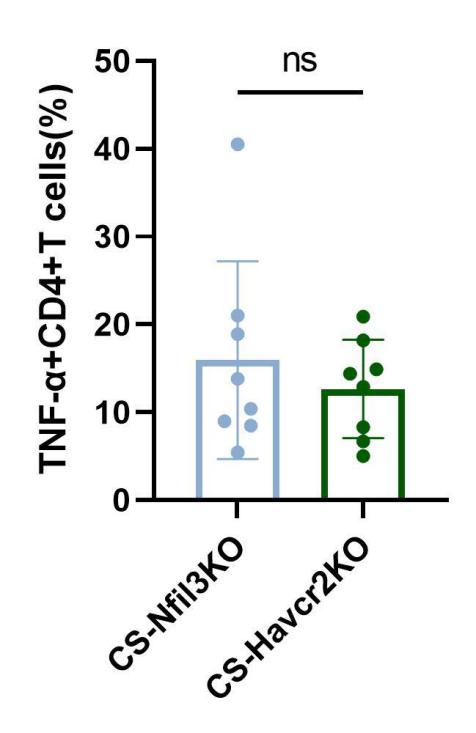


Supplementary Figure 15. Comparisons of TNF-α in CD4+T cells between the Nfil3-KO mice and Havcr2-KO COPD mice (n=8).The data, based on three independent experiments, are presented as means ± SD and differences between groups were analyzed using a non-parametric test (Student's t test).


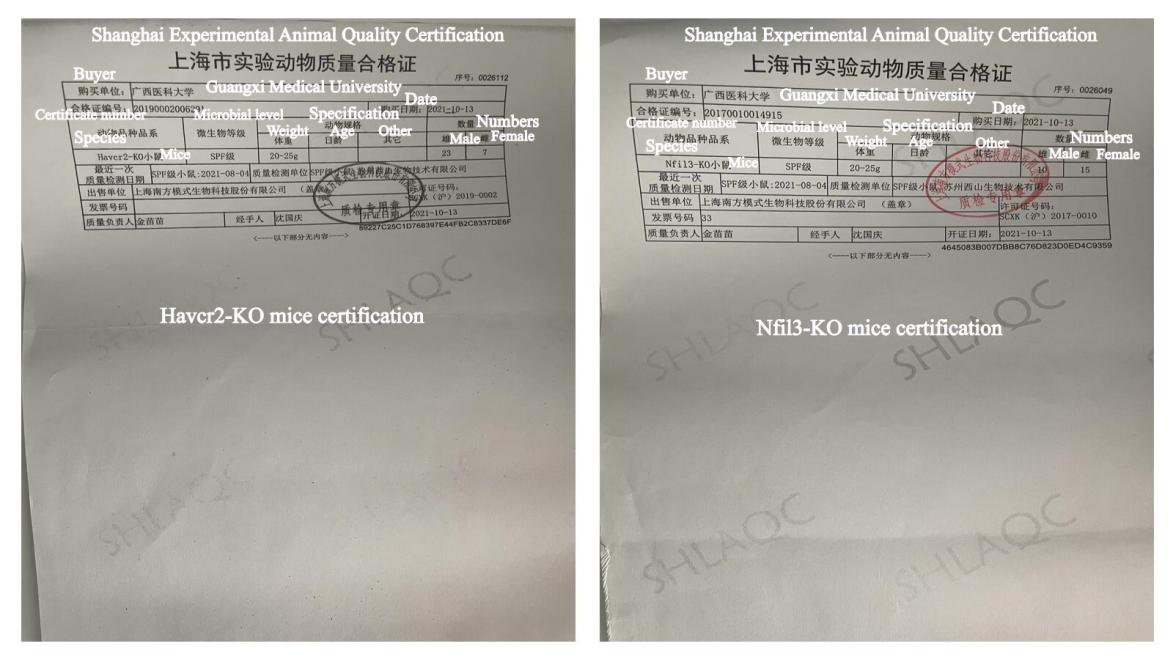


Supplementary Figure 16. Knockout mice certification (Havcr2 and Nfil3).
