# Supplementary material for: NFIL3/Tim3 axis regulates effector Th1 inflammation in COPD mice
Source: Front Immunol. 2024 Nov 1;15:1482213. doi: 10.3389/fimmu.2024.1482213 (PMC11563780; doi:10.3389/fimmu.2024.1482213)
Supplement: Supplementary file 1 [file DataSheet1.zip › Supplementary Materials/Supplementary Material .Bioinformatic Codes.docx]

#整合10X单细胞数据读取并分离样本

rm(list=ls())

library(Seurat)

library(dplyr)

library(future)

library(future.apply)

library(dplyr)

library(msigdbr)

library(clusterProfiler)

library(data.table)

library(ggplot2)

library(patchwork)

library(Matrix)

options(future.globals.maxSize = 60000 * 1024^2)

getwd()

feature <- read.table(gzfile('features.tsv.gz'))

head(feature)

cellname <- read.table(gzfile('barcodes.tsv.gz'),

header = F,sep = '\t')[,1] %>% as.vector()

length(cellname)

cellname[1:5]

library(Matrix)

mymatrix <- readMM(gzfile('matrix.mtx.gz'))

mymatrix <- as(mymatrix,'CsparseMatrix')

colnames(mymatrix) <- cellname

rownames(mymatrix) <- feature$V1

scRNA <- CreateSeuratObject(counts = mymatrix,

project = "8sample", min.cells = 3, min.features = 200)

sample.num <- lapply(colnames(scRNA), function(x){strsplit(x,"__")[[1]][1]}) %>% unlist() %>% as.character()

unique(sample.num)

names(sample.num) <- colnames(scRNA)

scRNA[['sample.num']] <- sample.num

unique(scRNA$sample.num)

table(scRNA@meta.data$orig.ident)

#添加样本分组信息

metadata1 <- data.table::fread("group.csv",header = TRUE)

metadata <- FetchData(scRNA,"orig.ident")

metadata$cell_id <- rownames(metadata)

metadata <- left_join(x = metadata,y = metadata1,by = "orig.ident")

rownames(metadata) <- metadata$cell_id

scRNA <- AddMetaData(scRNA,metadata = metadata)

#开始正常Seurat流程

scRNA[["percent.mt"]] <- PercentageFeatureSet(scRNA, pattern = "^mt-")

scRNA <- subset(scRNA, subset = nFeature_RNA > 200 & nFeature_RNA < 2000 & percent.mt < 20)

scRNA <- NormalizeData(scRNA, normalization.method = "LogNormalize", scale.factor = 10000)

scRNA <- FindVariableFeatures(scRNA, selection.method = "vst", nfeatures = 2000)

scRNA <- ScaleData(scRNA, features = rownames(scRNA))

scRNA <- ScaleData(scRNA, verbose = FALSE)

scRNA <- RunPCA(scRNA, features = VariableFeatures(object = scRNA))

print(scRNA[["pca"]], dims = 1:5, nfeatures = 5)

scRNA <- FindNeighbors(scRNA,reduction = "pca",dims = 1:30)

scRNA <- RunUMAP(scRNA,reduction = "pca",dims = 1:30)

scRNA <- FindClusters(scRNA,resolution = 0.5)

p1<-DimPlot(scRNA,reduction = "umap",group.by = "group")

p2<-DimPlot(scRNA,reduction = "umap",shuffle = TRUE,label = TRUE,repel = TRUE)

p1+p2

#harmony去批次合并分析

library(Rcpp)

library(harmony)

scRNA<-RunHarmony(scRNA,group.by.vars = c("orig.ident"), plot_convergence = TRUE)

harmony_embeddings <- Embeddings(scRNA, 'harmony')

dim(harmony_embeddings)

p3 <- DimPlot(object = scRNA, reduction = "harmony", pt.size = .1, group.by = "orig.ident")

p4 <- VlnPlot(object = scRNA, features = "harmony_1", group.by = "orig.ident", pt.size = .1)

CombinePlots(plots=list(p3,p4))

scRNA<- scRNA %>%

RunUMAP(reduction = "harmony", dims = 1:50) %>%

RunTSNE(reduction = "harmony", dims = 1:50) %>%

FindNeighbors(reduction = "harmony", dims = 1:50)

scRNA<-FindClusters(scRNA,resolution = 0.5)

table(Idents(scRNA))

scRNA@meta.data[1:5,]

DimPlot(scRNA,shuffle = TRUE,reduction = "tsne",label = T)

DimPlot(scRNA,shuffle = TRUE,reduction = "tsne",group.by = "group")

DimPlot(scRNA,shuffle = TRUE,reduction = "tsne",split.by = "group")

#细胞marker鉴定

DefaultAssay(scRNA) <- "RNA"

scRNA <- JoinLayers(scRNA)

markers <- FindAllMarkers(scRNA,only.pos = TRUE,min.pct = 0.25,logfc.threshold = 0.25)

markers = markers[markers$p_val_adj<=0.05,]

write.csv(markers,"./scRNA.markers.csv")

markers_table=markers%>%

group_by(cluster)%>%

top_n(n=5,wt=avg_log2FC)

FeaturePlot(scRNA,features = markers_table[markers_table$cluster==0,]$gene,min.cutoff = "q10")#UMAP图

VlnPlot(scRNA,,features = markers_table[markers_table$cluster==0,]$gene)#小提琴图

DotPlot(scRNA,,features = markers_table[markers_table$cluster==0,]$gene,cols = c("blue","red"),dot.scale=8)+RotatedAxis()#气泡图

scRNA<-ScaleData(scRNA,features = unique(markers_table$gene))#top基因

DoHeatmap(scRNA,features = markers_table$gene,size = 3,draw.lines = FALSE)#热图

cluster.averages<-AverageExpression(scRNA,return.seurat = TRUE)

QC1=DoHeatmap(object = cluster.averages,features = markers_table$gene,size = 3,draw.lines = FALSE)

ggsave("Z-QC1.pdf",QC1,width = 10,height = 17)

#显示感兴趣基因

DotPlot(scRNA, features = c("Cd3e"))

#绘制各marker基因表达小提琴图

library(patchwork) # 拼图

library(ggplot2) # 绘图

library(Seurat) # readRDS, VlnPlot

library(scales) # show_col

library(ggsci)

cors<-pal_igv()(12)

features <- c("Epcam", "Cd3e", "Vwf","Col1a1","Cd79a", "Cd14","Cpa3","Upk3b")

p2 <- VlnPlot(scRNA, features, stack = TRUE,

sort = TRUE, flip = TRUE, cols = cors) +

theme(legend.position = "none")

p2

#细胞注释

Idents(scRNA)=scRNA$seurat_clusters

scRNA<-RenameIdents(scRNA,`0`="endothelial cell",`1`="endothelial cell",`2`="myeloid",`3`="endothelial cell",`4`="B cell",

`5`="mast cell",`6`="Tcell",`7`="endothelial cell",`8`="fibroblast",`9`="endothelial cell",

`10`="epithelial cell",`11`="NK",`12`="pericyte",`13`="myeloid",`14`="myeloid",

`15`="SLC16A7+ cell",`16`="epithelial cell",`17`="myeloid",`18`="fibroblast",

`19`="epithelial cell",`20`="epithelial cell",`21`="Tcell",`22`="mesothelial cell",`23`="red blood cell",

`24`="Tcell",`25`="endothelial cell")

DimPlot(scRNA,shuffle = TRUE,reduction = "tsne",label = T)

DimPlot(scRNA,shuffle = TRUE,reduction = "tsne",split.by = "group")

scRNA$seurat_clusters<-Idents(scRNA)

scRNA@meta.data[1:5,]

saveRDS(scRNA,file="./data1.rds")#存一下

#计算组间差异基因

data<-readRDS("./data1.rds")

data$celltype.group <- paste(data$seurat_clusters, data$group, sep = "_")

data$celltype <- Idents(data)

Idents(data) <- "celltype.group"

mydeg <- FindMarkers(data,ident.1 = 'Tcell_CS',ident.2 = 'Tcell_Air', verbose = TRUE, test.use = 'wilcox',min.pct = 0.1)

head(mydeg)

write.csv(mydeg,file="./Z-T-markers-CS-Air.csv")

#提取T进一步分析

Tcell<-data[,Idents(data)%in%c("Tcell")]

rm(data)

gc()

s.genes <- cc.genes$s.genes

g2m.genes <- cc.genes$g2m.genes

Tcell <- CellCycleScoring(Tcell, s.features = s.genes, g2m.features = g2m.genes, set.ident = TRUE)

Tcell@meta.data[1:5,]

Tcell<-NormalizeData(Tcell,verbose = T)

Tcell<-FindVariableFeatures(Tcell,selection.method = "vst", nfeatures = 2000)

Tcell<-ScaleData(Tcell,vars.to.regress = c("percent.mt","S.Score","G2M.Score"),verbose = FALSE)

Tcell<-RunPCA(Tcell,npcs = 50)

ElbowPlot(Tcell,ndims = 50)

p1 <- DimPlot(object = Tcell, reduction = "pca", pt.size = .1, group.by = "orig.ident")

p2 <- VlnPlot(object = Tcell, features = "PC_1", group.by = "orig.ident", pt.size = .1)

CombinePlots(plots=list(p1,p2))

library(harmony)

Tcell<-RunHarmony(Tcell,"orig.ident", plot_convergence = TRUE)

harmony_embeddings <- Embeddings(Tcell, 'harmony')

dim(harmony_embeddings)

p3 <- DimPlot(object = Tcell, reduction = "harmony", pt.size = .1, group.by = "orig.ident")

p4 <- VlnPlot(object = Tcell, features = "harmony_1", group.by = "orig.ident", pt.size = .1)

CombinePlots(plots=list(p3,p4))

Tcell <- Tcell %>%

RunUMAP(reduction = "harmony", dims = 1:50) %>%

RunTSNE(reduction = "harmony", dims = 1:50) %>%

FindNeighbors(reduction = "harmony", dims = 1:50)

Tcell<-FindClusters(Tcell,resolution = 0.75)

DimPlot(Tcell,shuffle = TRUE,reduction = "tsne",label = TRUE)

DimPlot(Tcell,shuffle = TRUE,reduction = "tsne",split.by = "group")

DimPlot(Tcell,shuffle = TRUE,reduction = "tsne",group.by = "group")

#细胞marker鉴定

DefaultAssay(Tcell) <- "RNA"

Tcell <- JoinLayers(Tcell)

Tmarkers <- FindAllMarkers(Tcell,only.pos = TRUE,min.pct = 0.25,logfc.threshold = 0.25)

Tmarkers = Tmarkers[Tmarkers$p_val_adj<=0.05,]

write.csv(Tmarkers,"./Tmarkers.csv")

Tmarkers_table=Tmarkers%>%

group_by(cluster)%>%

top_n(n=5,wt=avg_log2FC)

FeaturePlot(Tcell,features = Tmarkers_table[Tmarkers_table$cluster==0,]$gene,min.cutoff = "q10")#UMAP图

VlnPlot(Tcell,,features = Tmarkers_table[Tmarkers_table$cluster==0,]$gene)#小提琴图

DotPlot(Tcell,,features = Tmarkers_table[Tmarkers_table$cluster==0,]$gene,cols = c("blue","red"),dot.scale=8)+RotatedAxis()#气泡图

Tcell<-ScaleData(Tcell,features = unique(Tmarkers_table$gene))#top5基因

DoHeatmap(Tcell,features = Tmarkers_table$gene,size = 3,draw.lines = FALSE)#热图

cluster.averages<-AverageExpression(Tcell,return.seurat = TRUE)

DoHeatmap(object = cluster.averages,features = Tmarkers_table$gene,size = 3,draw.lines = FALSE)

#显示感兴趣基因

DotPlot(Tcell, features = c("Gzma", "Sell","Ccr8","Tnfrsf4","Eomes","Zbtb16","Cd4"))

features <- c("Gzma", "Sell","Ccr8","Tnfrsf4","Eomes","Zbtb16","Cd4")

p2 <- VlnPlot(Tcell, features, stack = TRUE,

sort = TRUE, flip = TRUE, cols = cors) +

theme(legend.position = "none")

p2

saveRDS(Tcell,file="./Tdata1.rds")#存一下

Tcell<-readRDS("./Tdata1.rds")

#细胞注释

Idents(Tcell)=Tcell$seurat_clusters

Tcell<-RenameIdents(Tcell,`0`="CD4 Tn",`1`="CD8 Teff",`2`="CD4 Teff",`3`="Tem",`4`="NKT",`5`="undefined",`6`="Treg")

DimPlot(Tcell,shuffle = TRUE,reduction = "tsne",label = TRUE,repel = TRUE)

DimPlot(Tcell,split.by = "group",shuffle = TRUE,label = TRUE,repel = TRUE)

#新矩阵添加细胞亚群信息

Tcell$Seurat_harmony<-Idents(Tcell)

Tcell@meta.data[1:5,]

saveRDS(Tcell,file="./Tdata1.rds")#存一下

#绘制基因表达密度图

Tcell<-readRDS("./Tdata1.rds")

library(Nebulosa)

plot_density(Tcell,"Il27ra")

plot_density(Tcell,c("Tbx21","Cd4"),joint = TRUE)

#拟时序分析

library(monocle)

#载入注释后的数据

pbmc<-readRDS("./Tdata1.rds")

pbmc

#pbmc<-Tcell[,Idents(Tcell)%in%c("CD4 Tn","CD4 Teff")]

pbmc<-pbmc[,Idents(pbmc)%in%c("CD4 Tn","CD4 Teff")]

#Extract data, phenotype data, and feature data from the SeuratObject

pbmc1 = GetAssayData(object=pbmc, slot="data", assay=DefaultAssay(pbmc))

data <- as(as.matrix(pbmc1), 'sparseMatrix')

pd <- new('AnnotatedDataFrame', data = pbmc@meta.data)

fData <- data.frame(gene_short_name = row.names(data), row.names = row.names(data))

fd <- new('AnnotatedDataFrame', data = fData)

#构建S4对象，CellDataSet

HSMM <- newCellDataSet(data,

phenoData = pd,

featureData = fd,

lowerDetectionLimit = 0.01,

expressionFamily = negbinomial.size())

HSMM <- estimateSizeFactors(HSMM)

HSMM <- estimateDispersions(HSMM)

HSMM <- detectGenes(HSMM, min_expr = 3 )

print(head(fData(HSMM)))

expressed_genes <- row.names(subset(fData(HSMM),

num_cells_expressed >= 10))

head(pData(HSMM))

diff_test_res <- differentialGeneTest(HSMM[expressed_genes,],

fullModelFormulaStr = "~ seurat_clusters")

ordering_genes <- row.names (subset(diff_test_res, qval < 0.01)) ## 不要也写0.1 ，而是要写0.01。

HSMM <- setOrderingFilter(HSMM, ordering_genes)

plot_ordering_genes(HSMM)

HSMM <- reduceDimension(HSMM, max_components = 5,

num_dim = 20,

method = 'DDRTree') # DDRTree方式

HSMM <- orderCells(HSMM)

colour=c("#FF6A6A","#66CD00","#20B2AA","#FFA500","#9370DB","#1E90FF","#7CFC00","#FFFF00",

"#808000","#FF00FF","#FA8072","#7B68EE","#9400D3","#800080","#A0522D","#D2B48C","#D2691E","#87CEEB","#40E0D0","#5F9EA0",

"#FF1493","#0000CD","#008B8B","#FFE4B5","#8A2BE2","#228B22","#E9967A","#4682B4","#32CD32","#F0E68C","#FFFFE0","#EE82EE",

"#FF6347","#6A5ACD","#9932CC","#8B008B","#8B4513","#DEB887")

a1 <- plot_cell_trajectory(HSMM, color_by = "Seurat_harmony") + scale_color_manual(values = colour)

a1

a2 <- plot_cell_trajectory(HSMM, color_by = "State") + scale_color_manual(values = colour)

a2

a3 <- plot_cell_trajectory(HSMM, color_by = "Pseudotime")

a3

a4 <- plot_cell_trajectory(HSMM, markers = c("Nfil3"),use_color_gradient = T,show_branch_points = F)

a4

#目的基因趋势变化

to_be_tested <- row.names(subset(fData(HSMM), gene_short_name %in% c("Nfil3")))

cds_subset <- HSMM[to_be_tested,]

plot_genes_in_pseudotime(cds_subset, color_by = "Seurat_harmony") + scale_color_manual(values = colour)

p2 <- plot_complex_cell_trajectory(HSMM, x = 1, y = 2,

color_by = "Seurat_harmony")+

scale_color_manual(values = colour) +

theme(legend.title = element_blank())

p2
