# Supplementary material for: NFIL3/Tim3 axis regulates effector Th1 inflammation in COPD mice
Source: Front Immunol. 2024 Nov 1;15:1482213. doi: 10.3389/fimmu.2024.1482213 (PMC11563780; doi:10.3389/fimmu.2024.1482213)
Supplement: Supplementary file 1 [file DataSheet1.zip › Supplementary Materials/Supplementary Table 5.reagents.docx]

# 1.CD4 Monoclonal Antibody (GK1.5), FITC, eBioscience™；

# 2.CD4 Monoclonal Antibody (GK1.5), APC-eFluor™ 780, eBioscience™；

# 3.CD366 (TIM3) Monoclonal Antibody (RMT3-23), PE, eBioscience™；

# 4.IFN gamma Monoclonal Antibody (XMG1.2), APC, eBioscience™；

# 5.IFN gamma Monoclonal Antibody (XMG1.2), PE-Cyanine7, eBioscience™；

# 6.TNF alpha Monoclonal Antibody (MP6-XT22), PE-Cyanine7, eBioscience™；

# 7.TNF alpha Monoclonal Antibody (MP6-XT22), PerCP-eFluor™ 710, eBioscience™；

# 8.TNF alpha Monoclonal Antibody (MP6-XT22), eFluor™ 450, eBioscience™;

# 9.CD44 Monoclonal Antibody (IM7), APC-eFluor™ 780, eBioscience™；

# 10.CD44 Monoclonal Antibody (IM7), APC, eBioscience™；

# 11.CD62L (L-Selectin) Monoclonal Antibody (MEL-14), PE, eBioscience™；

# 12.CD62L (L-Selectin) Monoclonal Antibody (MEL-14), APC, eBioscience™；

# 13.T-bet Monoclonal Antibody (eBio4B10 (4B10)), PE-Cyanine7, eBioscience™；

# 14.E4BP4 (NFIL3) Monoclonal Antibody (S2M-E19), Alexa Fluor™ 647, eBioscience™；

# 15.CD3 Monoclonal Antibody (17A2), PerCP-eFluor™ 710, eBioscience™
