# Supplementary material for: NFIL3/Tim3 axis regulates effector Th1 inflammation in COPD mice
Source: Front Immunol. 2024 Nov 1;15:1482213. doi: 10.3389/fimmu.2024.1482213 (PMC11563780; doi:10.3389/fimmu.2024.1482213)
Supplement: Supplementary file 1 [file DataSheet1.zip › Supplementary Materials/Supplementary Table 6.Flow cytometry-templet.docx]

**Wild type mice:**

T1:

CD4: FITC

Tim3: PE

IFN-γ: PE-Cyanine7

T2:

CD4: FITC

Tim3: PE

TNF-α: PE-Cyanine7

T3:

CD4: FITC

Tim3: PE

CD44: APC-Cyanine7 (APC-eFluor^TM^780)

CD62L: APC

**Knock out mice:**

T1:

CD4: APC-Cyanine7 (APC-eFluor^TM^780)

CD44: APC

CD62L: PE

T2:

CD4: APC-Cyanine7 (APC-eFluor^TM^780)

IFN-γ: APC

TNF-α: Percp-Cyanine5.5 (PerCP-eFluor^TM^710)

T3:

CD4: APC-Cyanine7 (APC-eFluor^TM^780)

Tim3: PE

NFIL3: APC (Alexa Fluor^TM^647)

**In Vitro:**

T1:

CFSE: FITC

TNF-α: Percp-Cyanine5.5 (PerCP-eFluor^TM^710)

IFN-γ: PE-Cyanine7

T2:

CFSE: FITC

1. bet: PE-Cyanine7

NFIL3: APC (Alexa Fluor^TM^647)

**Nfil3-KO Tim3:**

CD3: Percp-Cyanine5.5 (PerCP-eFluor^TM^710)

CD4: FITC

Tim3: PE

**Supplementary Experiments:**

T3:

CD4: APC-Cyanine7 (APC-eFluor^TM^780)

Tim3: PE

NFIL3: APC (Alexa Fluor^TM^647)

T4:

CD4: APC-Cyanine7 (APC-eFluor^TM^780)

Tim3: PE

NFIL3: APC (Alexa Fluor^TM^647)

IFN-γ: PE-Cyanine7

TNF-α: V450 (eFluor^TM^450)
