# Supplementary figures and images for: NFIL3/Tim3 axis regulates effector Th1 inflammation in COPD mice
Source: Front Immunol. 2024 Nov 1;15:1482213. doi: 10.3389/fimmu.2024.1482213 (PMC11563780; doi:10.3389/fimmu.2024.1482213)

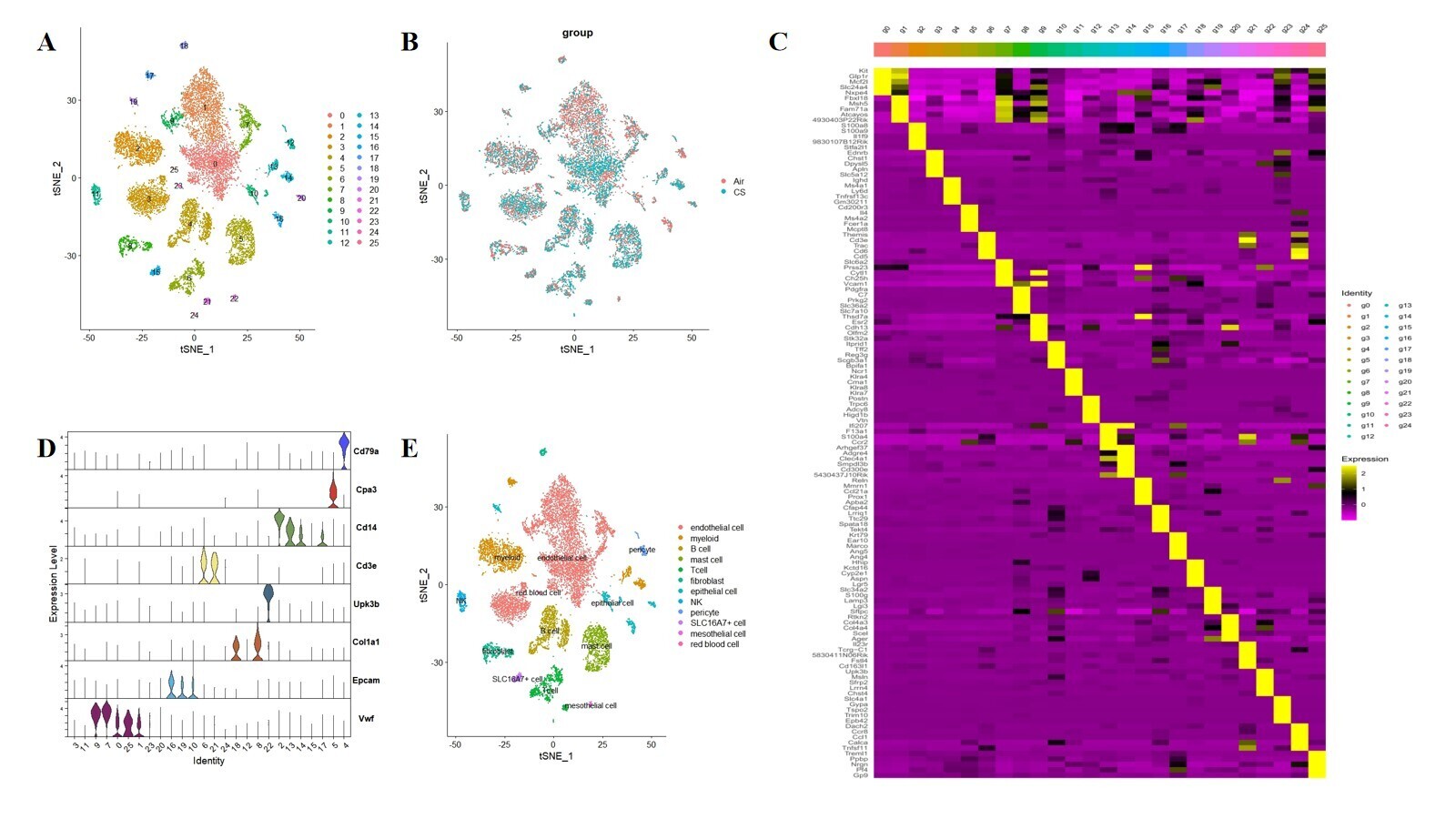

Supplement: Supplementary file 1 [file DataSheet1.zip › Supplementary Materials/SF1.jpg]

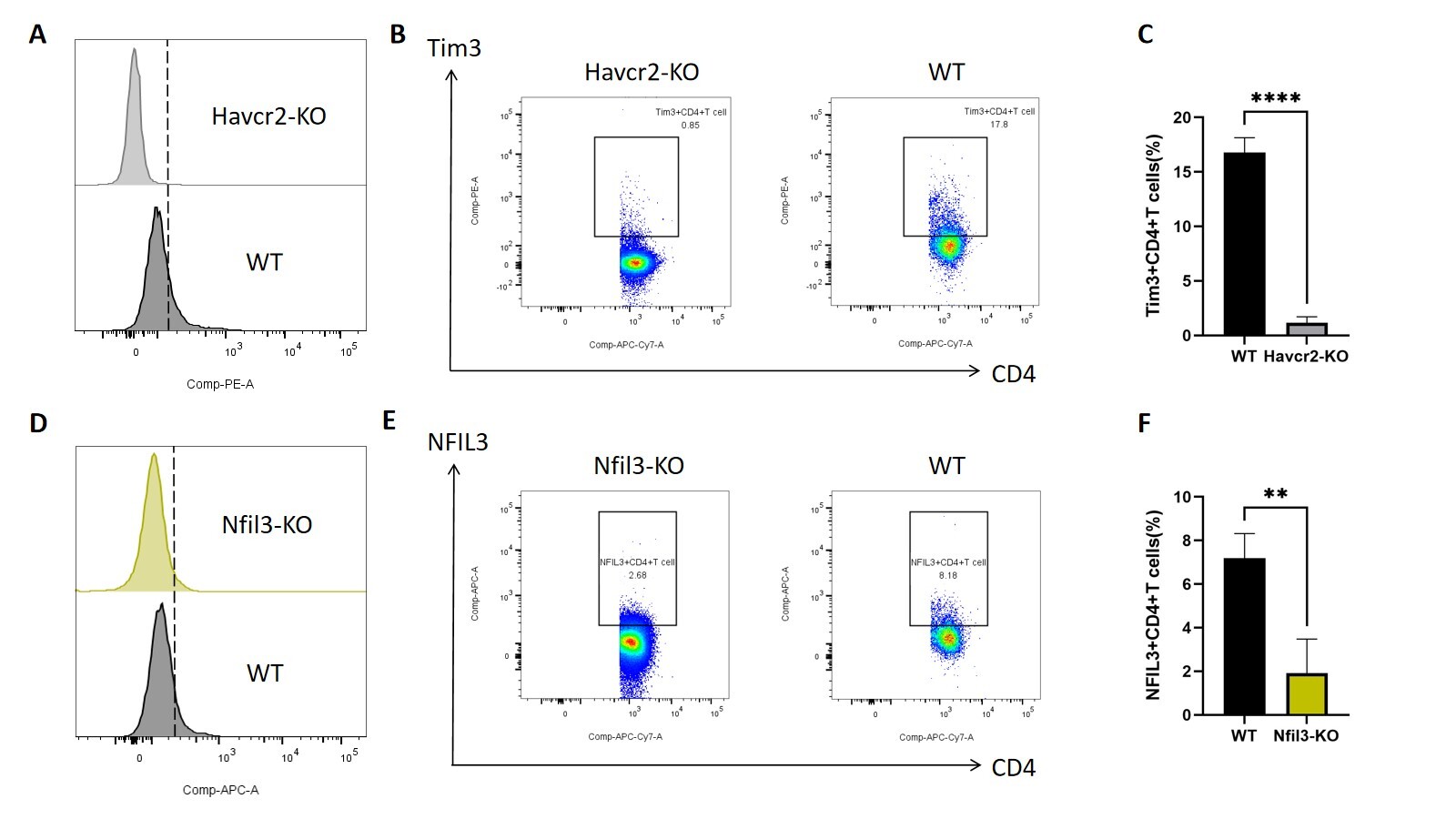

Supplement: Supplementary file 1 [file DataSheet1.zip › Supplementary Materials/SF10.jpg]

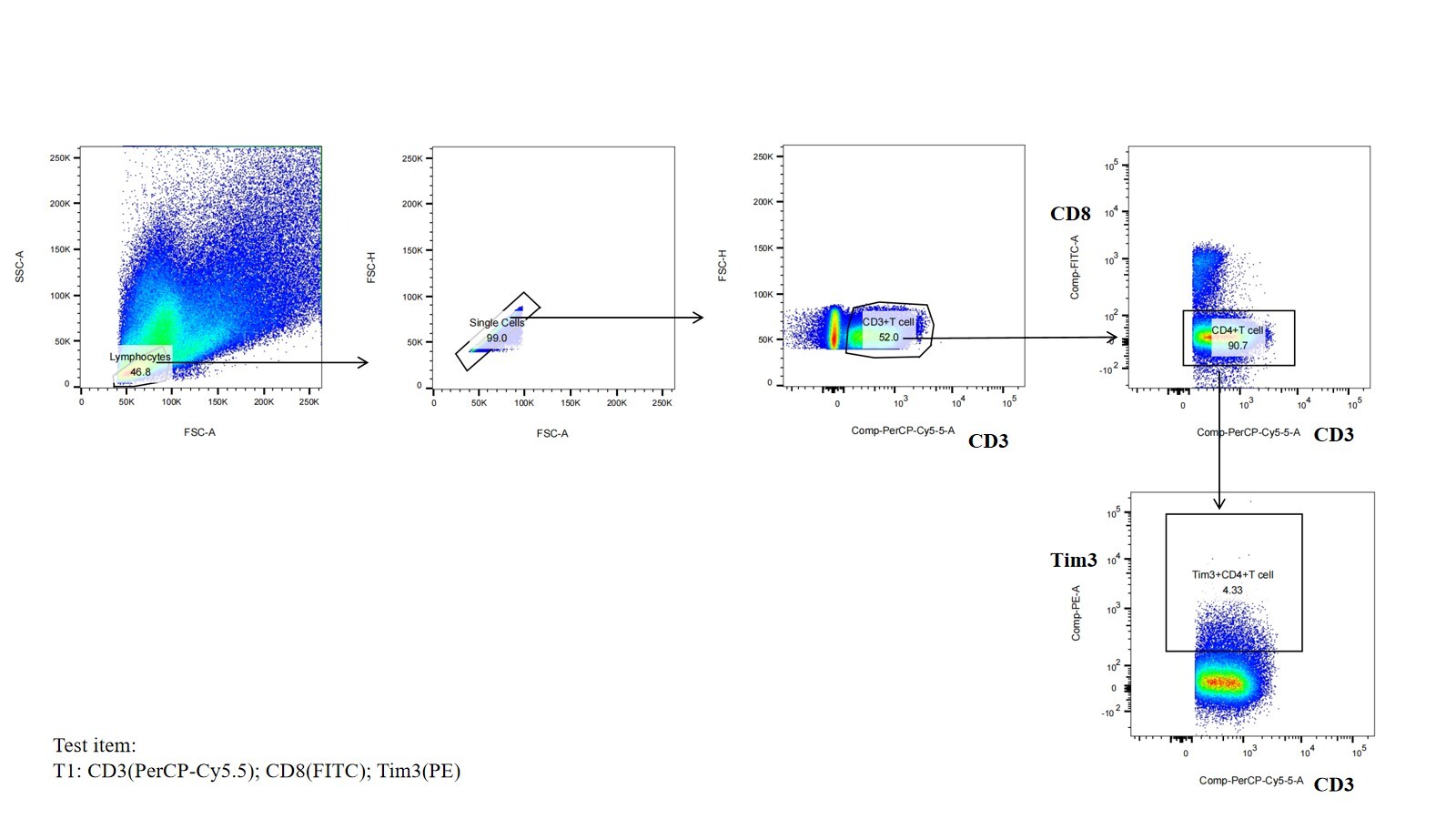

Supplement: Supplementary file 1 [file DataSheet1.zip › Supplementary Materials/SF11.jpg]

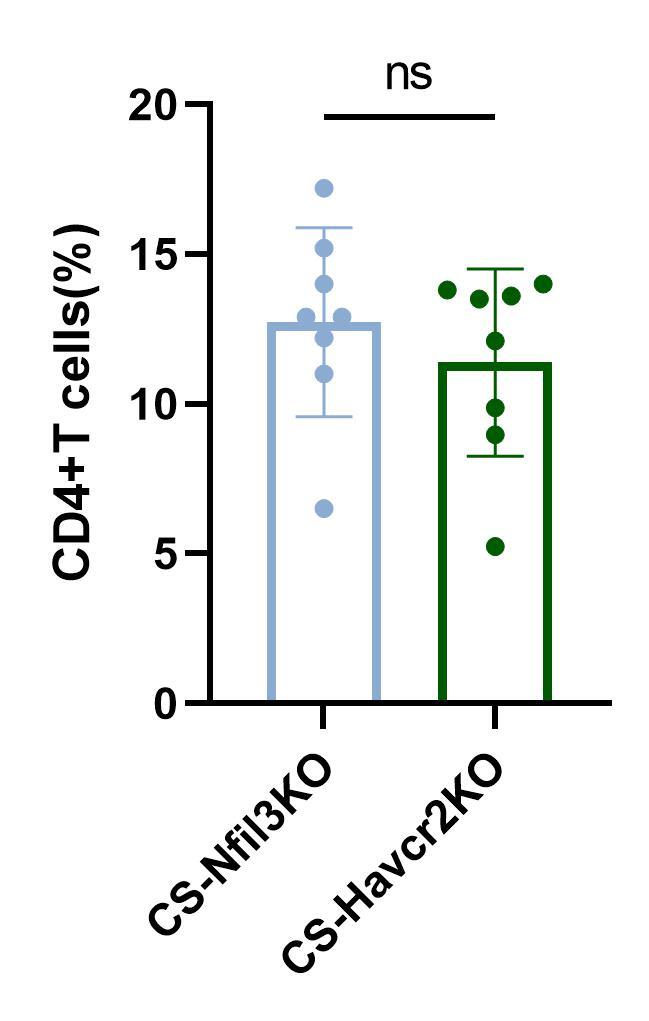

Supplement: Supplementary file 1 [file DataSheet1.zip › Supplementary Materials/SF12.jpg]

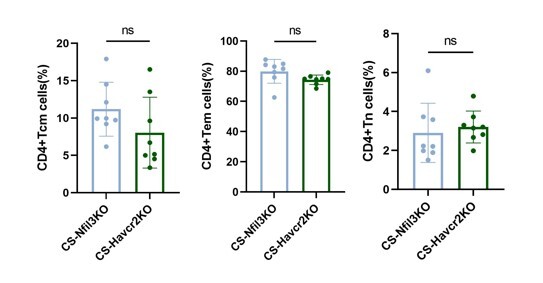

Supplement: Supplementary file 1 [file DataSheet1.zip › Supplementary Materials/SF13.jpg]

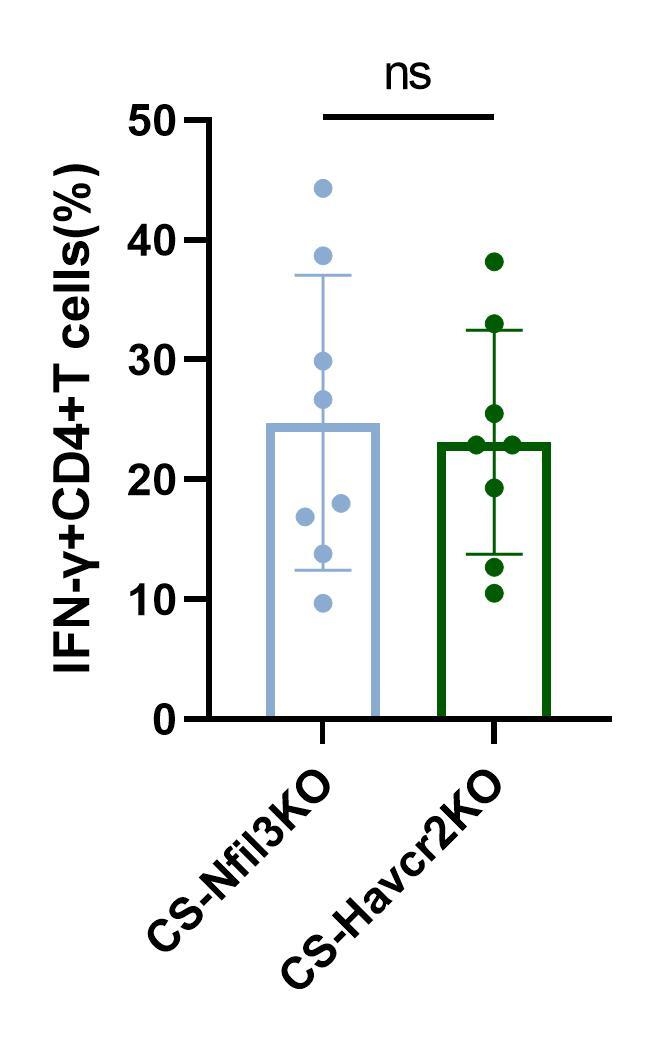

Supplement: Supplementary file 1 [file DataSheet1.zip › Supplementary Materials/SF14.jpg]

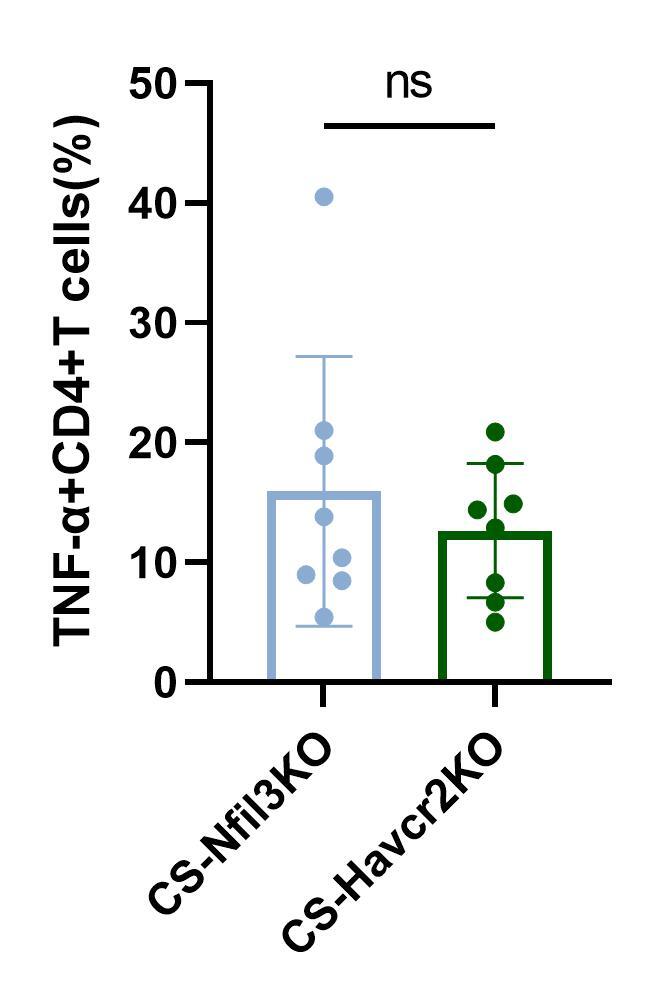

Supplement: Supplementary file 1 [file DataSheet1.zip › Supplementary Materials/SF15.jpg]

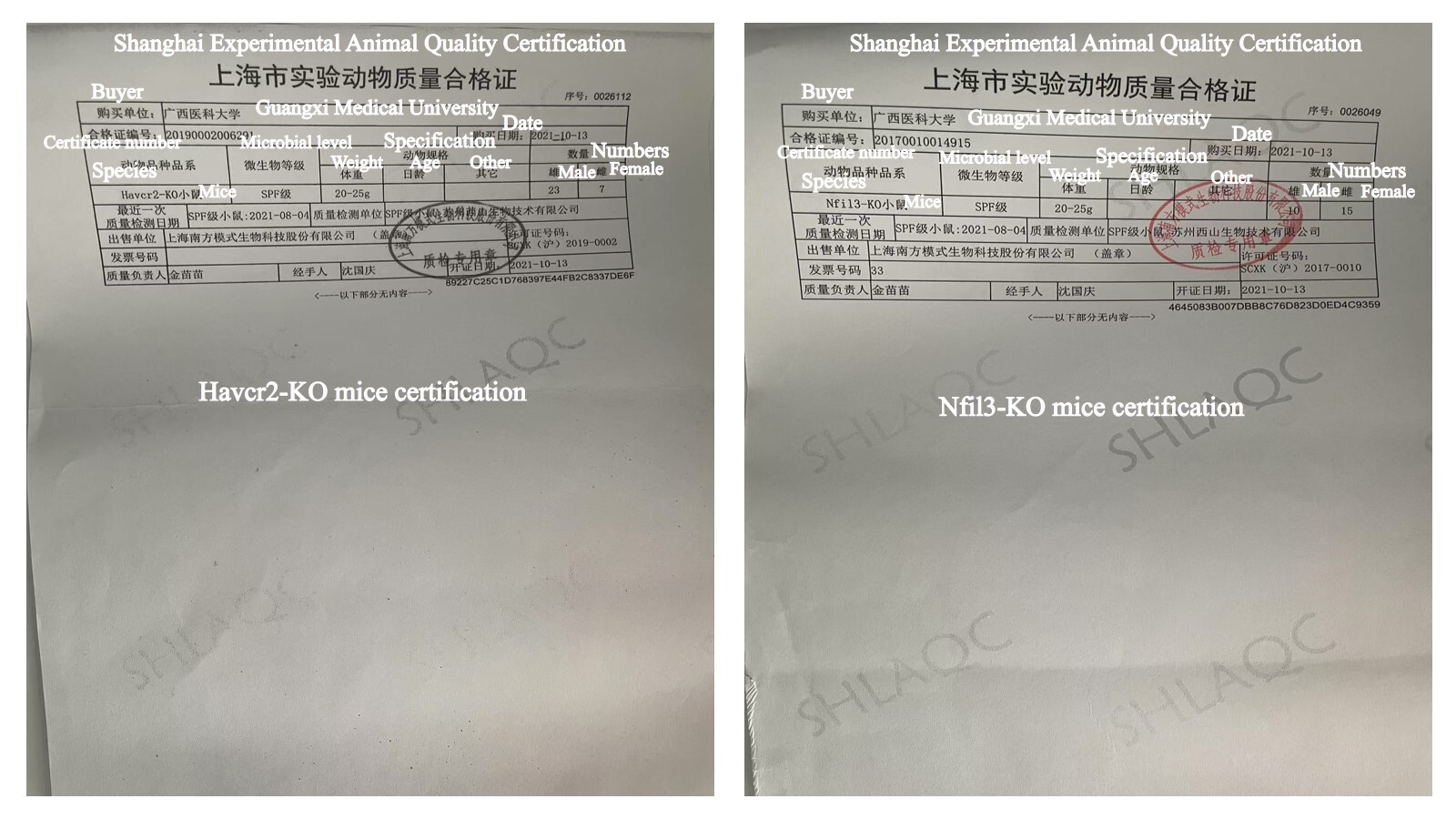

Supplement: Supplementary file 1 [file DataSheet1.zip › Supplementary Materials/SF16.jpg]

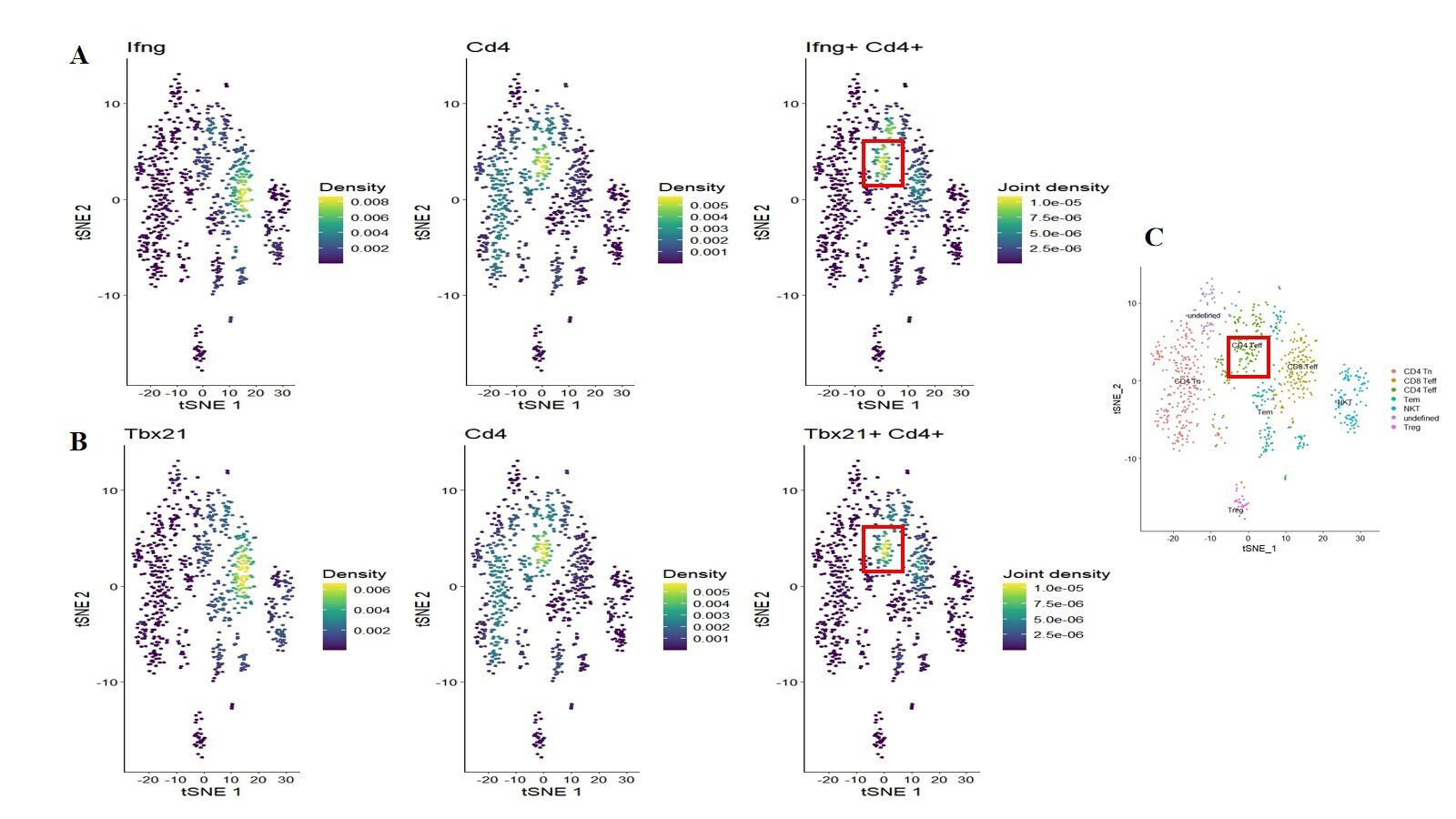

Supplement: Supplementary file 1 [file DataSheet1.zip › Supplementary Materials/SF2.jpg]

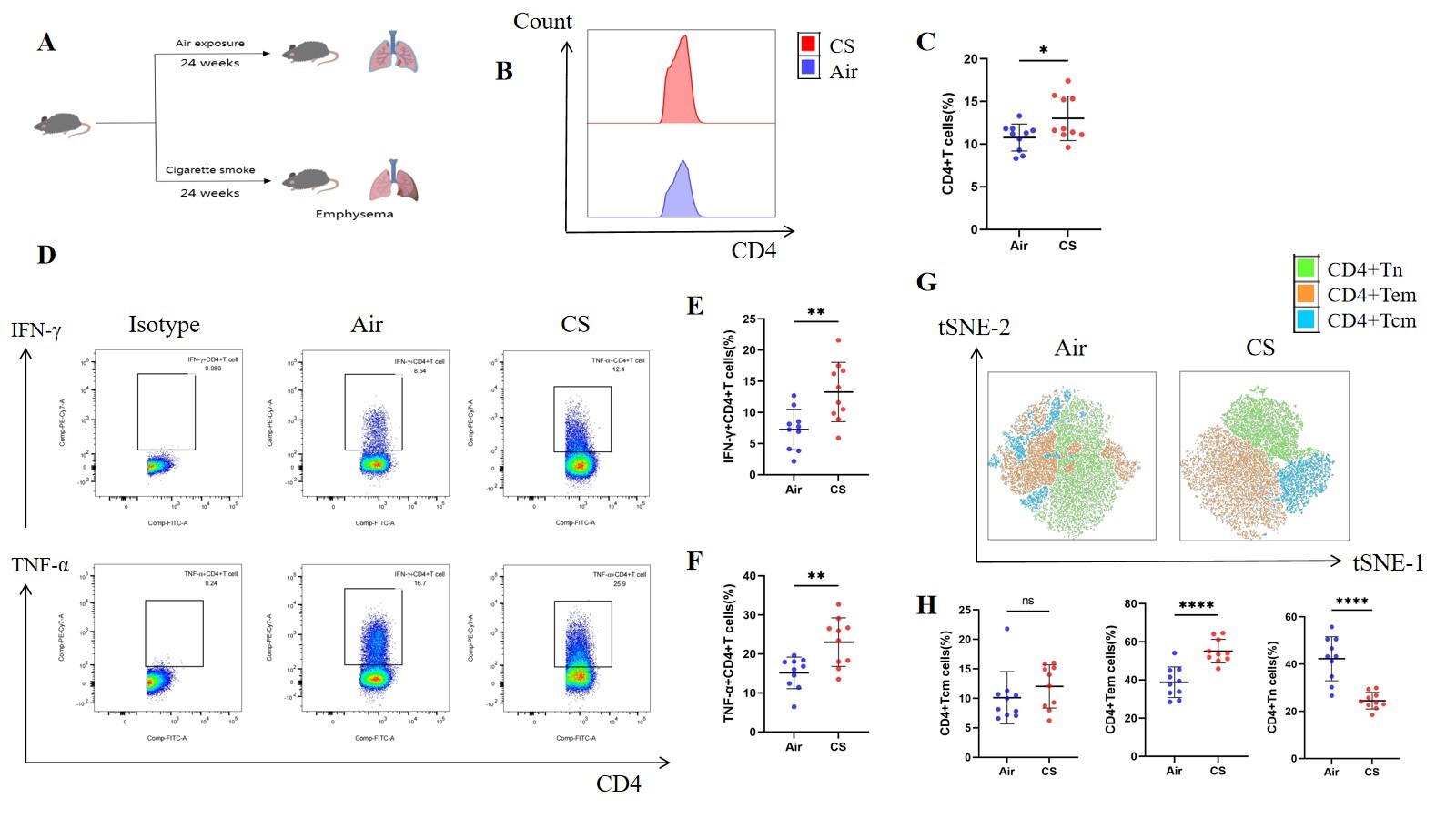

Supplement: Supplementary file 1 [file DataSheet1.zip › Supplementary Materials/SF3.jpg]

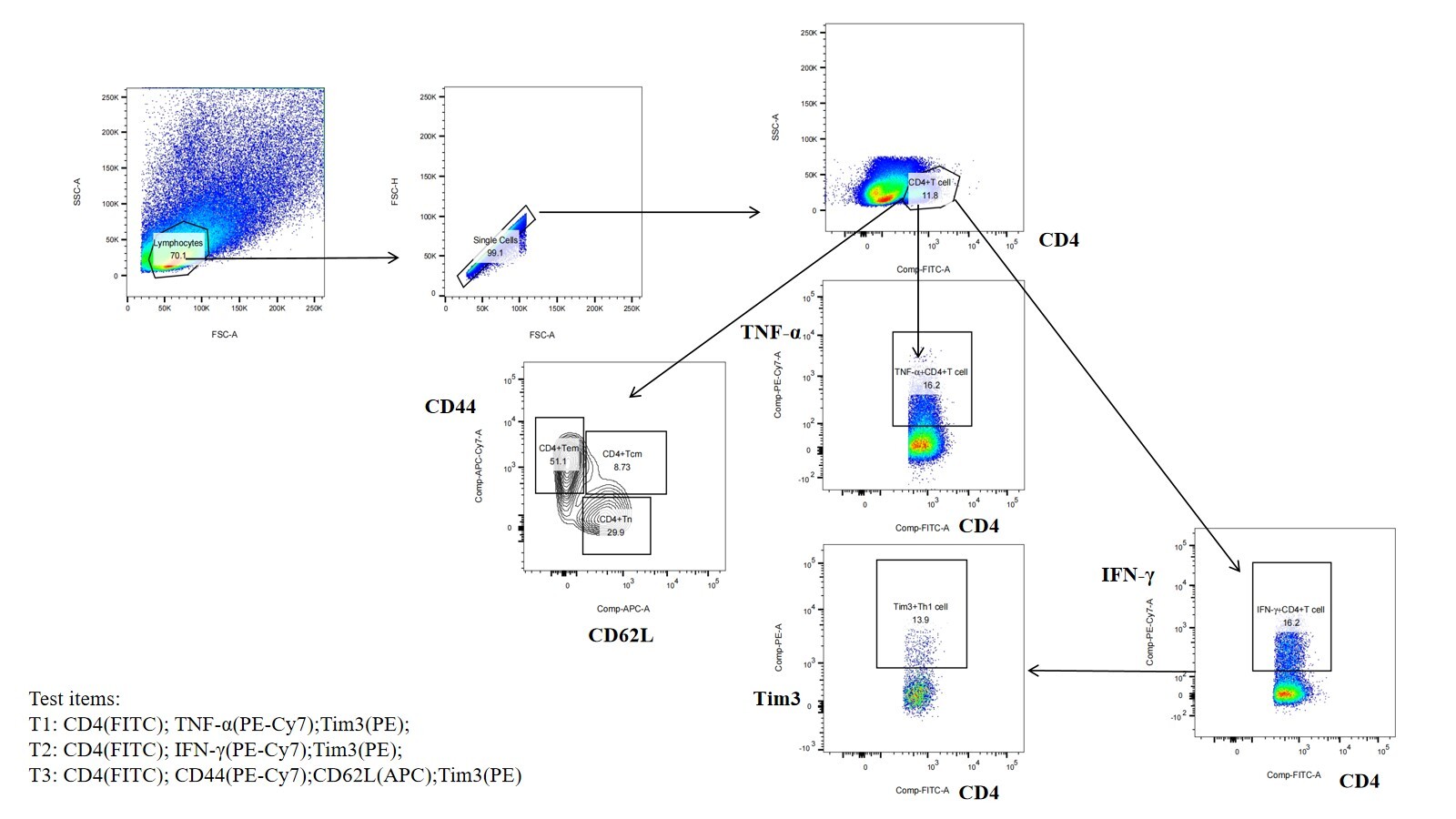

Supplement: Supplementary file 1 [file DataSheet1.zip › Supplementary Materials/SF4.jpg]

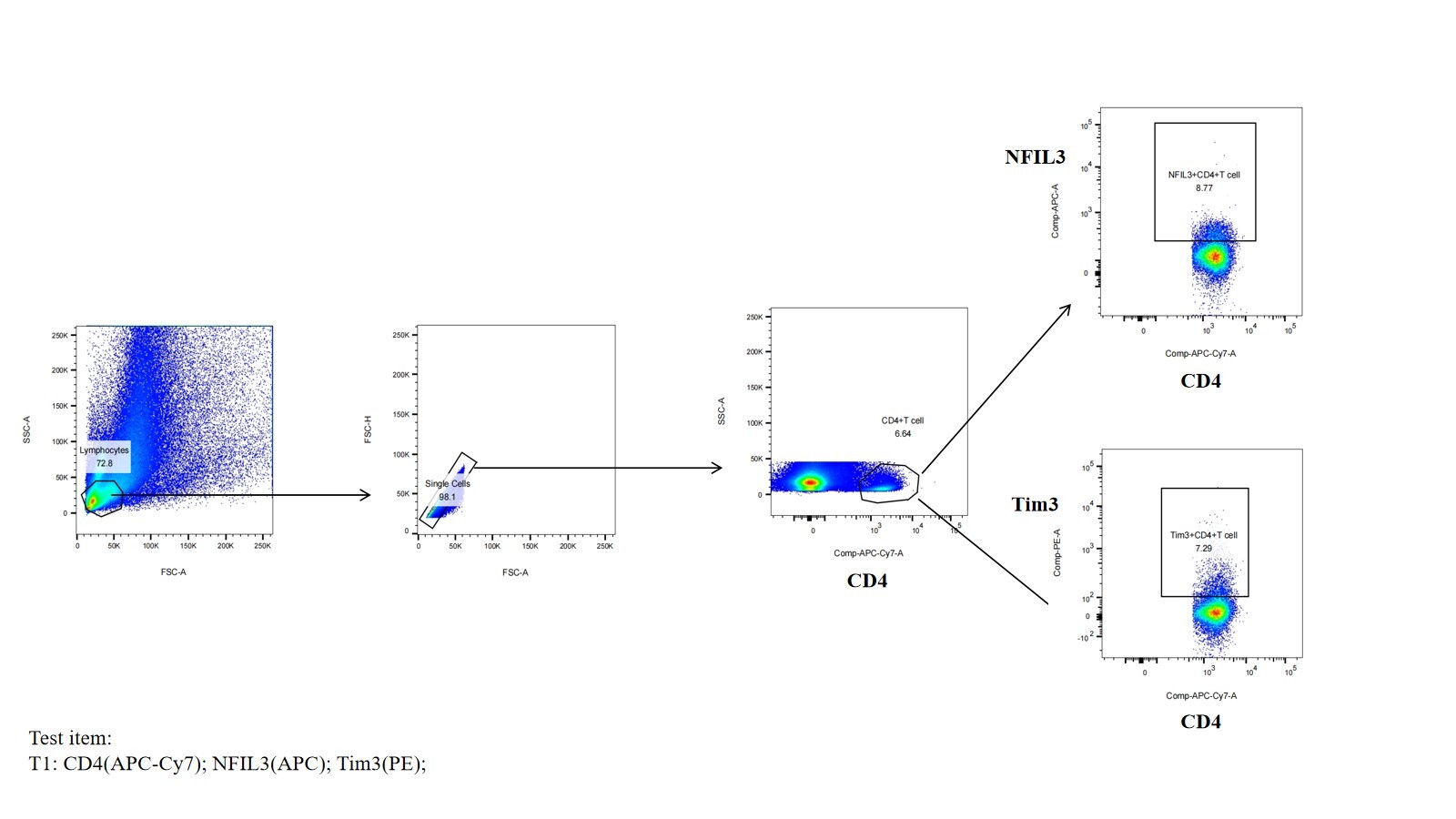

Supplement: Supplementary file 1 [file DataSheet1.zip › Supplementary Materials/SF5.jpg]

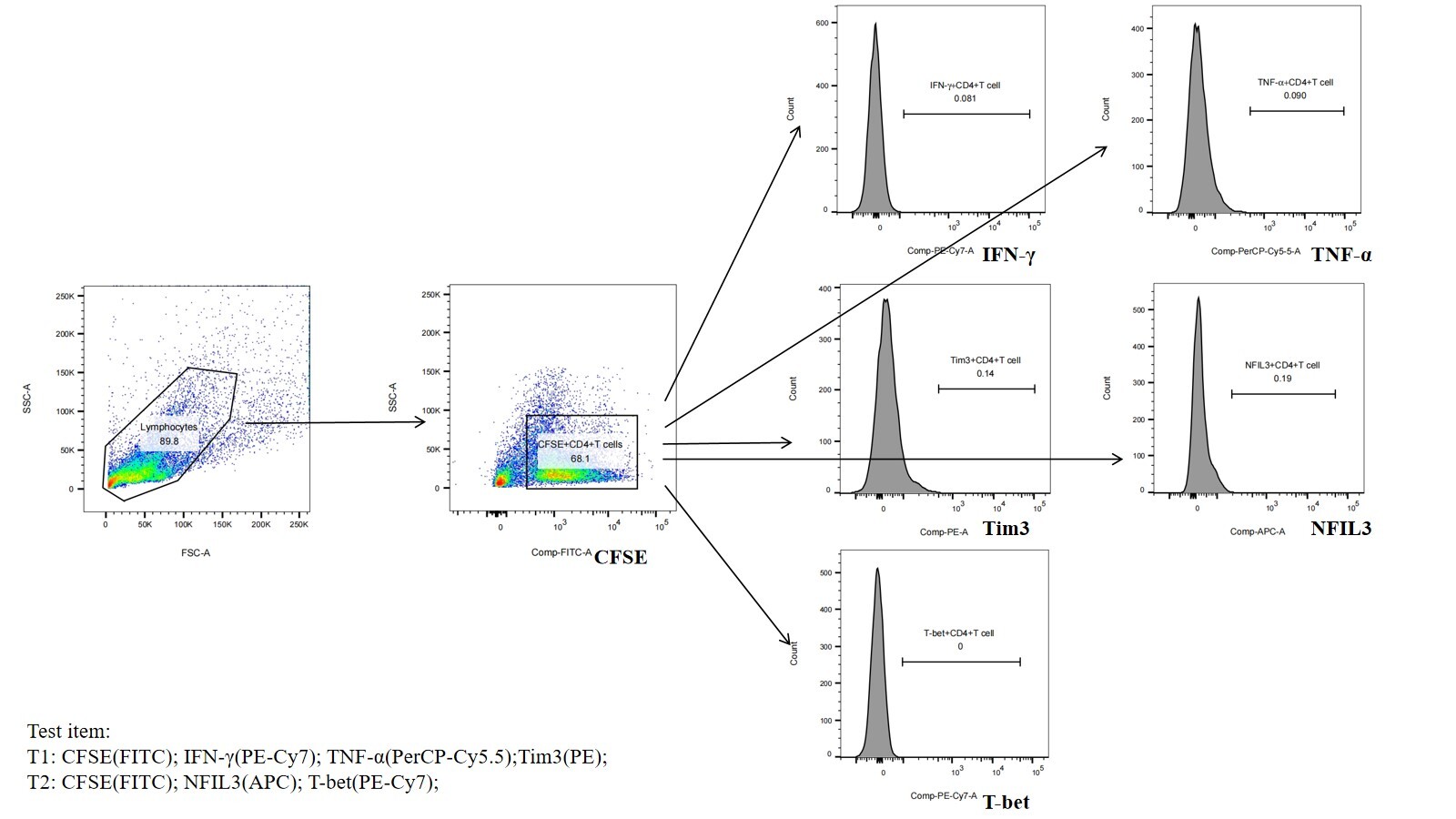

Supplement: Supplementary file 1 [file DataSheet1.zip › Supplementary Materials/SF6.jpg]

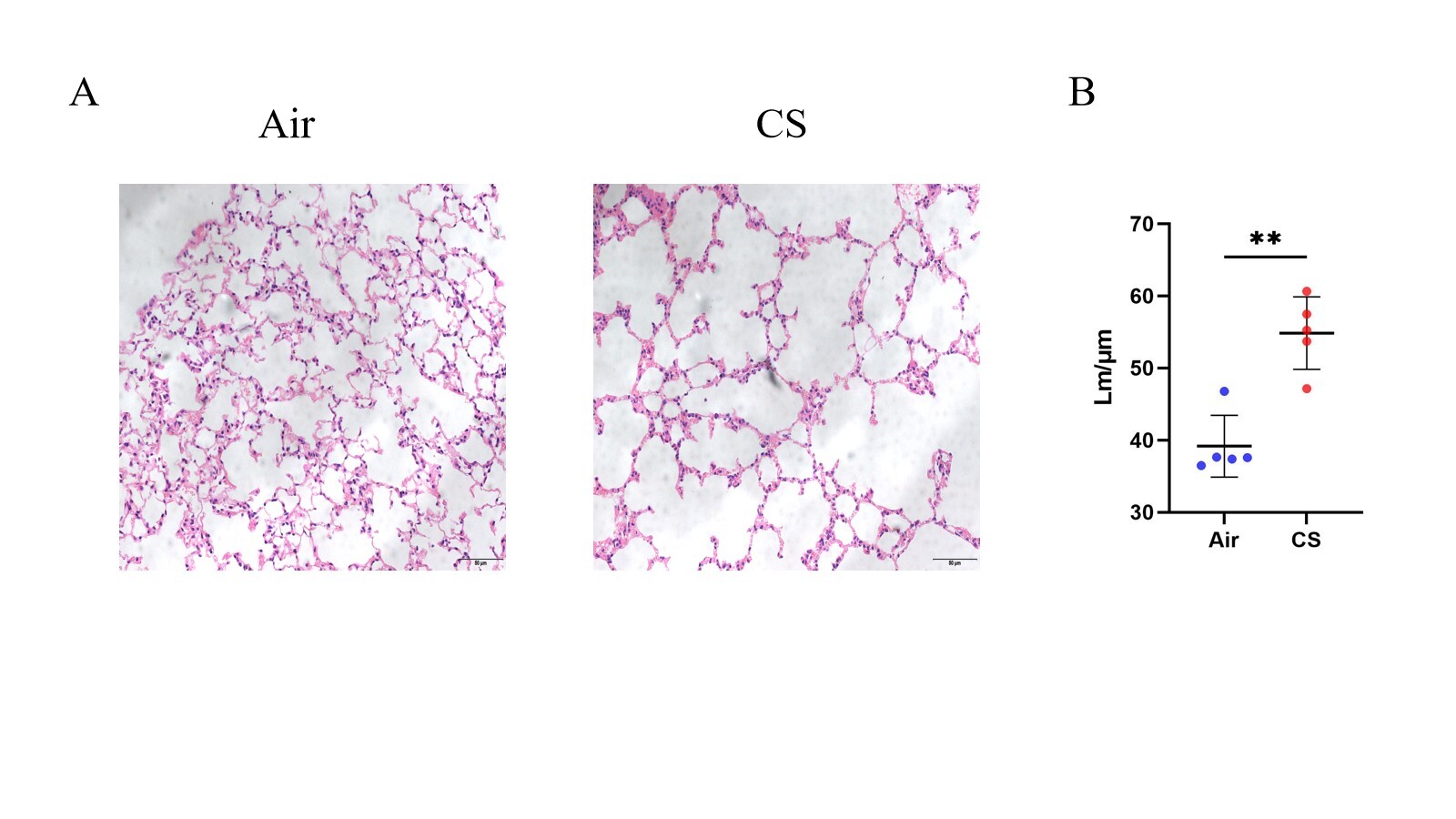

Supplement: Supplementary file 1 [file DataSheet1.zip › Supplementary Materials/SF7.jpg]

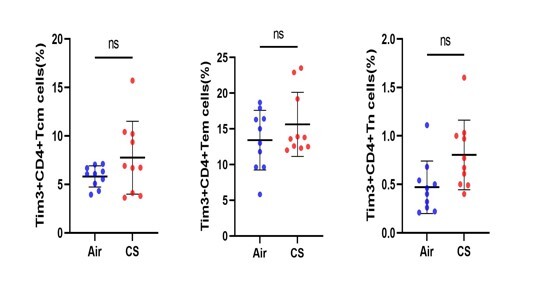

Supplement: Supplementary file 1 [file DataSheet1.zip › Supplementary Materials/SF8.jpg]

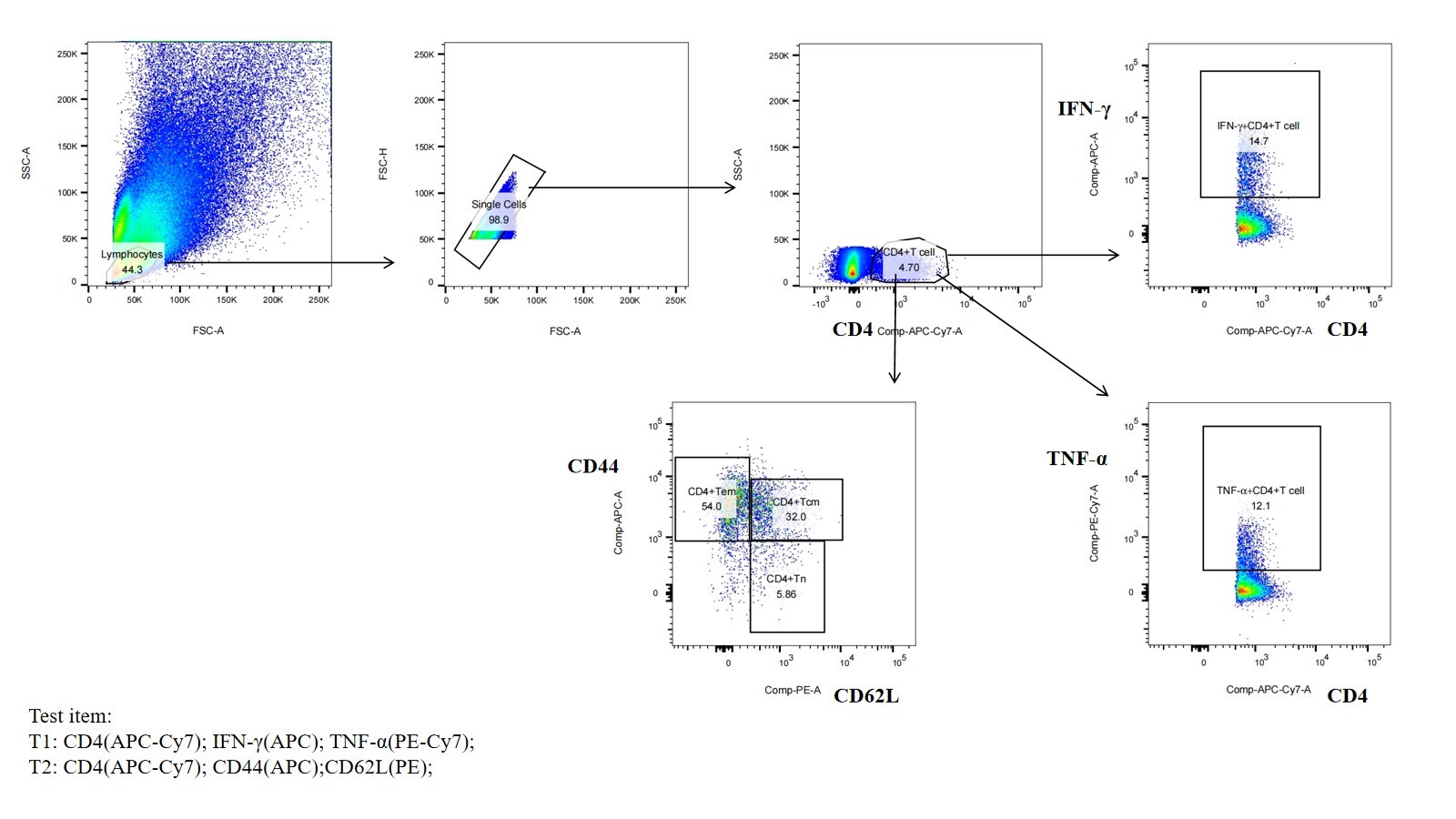

Supplement: Supplementary file 1 [file DataSheet1.zip › Supplementary Materials/SF9.jpg]
